# Crucial Role of the Chaperonin GroES/EL for Heterologous Production of the Soluble Methane Monooxygenase from *Methylomonas methanica* MC09

**DOI:** 10.1002/cbic.202200195

**Published:** 2022-04-29

**Authors:** Domenic Zill, Elisabeth Lettau, Christian Lorent, Franziska Seifert, Praveen K. Singh, Lars Lauterbach

**Affiliations:** ^1^ Technische Universität Berlin Institute for Chemistry Straße des 17. Juni 135 10437 Berlin Germany; ^2^ RWTH Aachen University iAMB - Institute of Applied Microbiology Worringer Weg 1 52074 Aachen Germany; ^3^ Martin-Luther-Universität Halle-Wittenberg Institut für Pharmazeutische Technologie und Biopharmazie Weinbergweg 22 06120 Halle (Saale) Germany

**Keywords:** biocatalysis, C1, green energy, hydroxylases, metalloenzymes

## Abstract

Methane is a widespread energy source and can serve as an attractive C1 building block for a future bioeconomy. The soluble methane monooxygenase (sMMO) is able to break the strong C−H bond of methane and convert it to methanol. The high structural complexity, multiplex cofactors, and unfamiliar folding or maturation procedures of sMMO have hampered the heterologous production and thus biotechnological applications. Here, we demonstrate the heterologous production of active sMMO from the marine *Methylomonas methanica* MC09 in *Escherichia coli* by co‐synthesizing the GroES/EL chaperonin. Iron determination, electron paramagnetic resonance spectroscopy, and native gel immunoblots revealed the incorporation of the non‐heme diiron centre and homodimer formation of active sMMO. The production of recombinant sMMO will enable the expansion of the possibilities of detailed studies, allowing for a variety of novel biotechnological applications.

## Graphical abstract

**Methane monooxygenase**: The chaperonin GroES/EL enables folding and correct assembly of soluble methane monooxygenase (sMMO) heterologously produced in *Escherichia coli*. The production of recombinant sMMO will enable a variety of novel biotechnological applications in future.

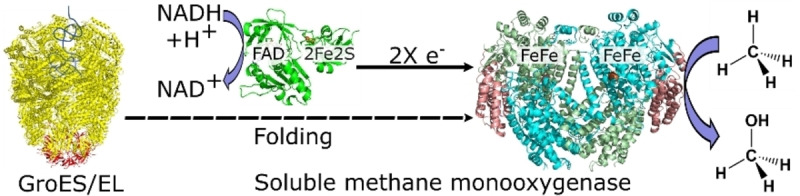

## Introduction

**Figure 1:**
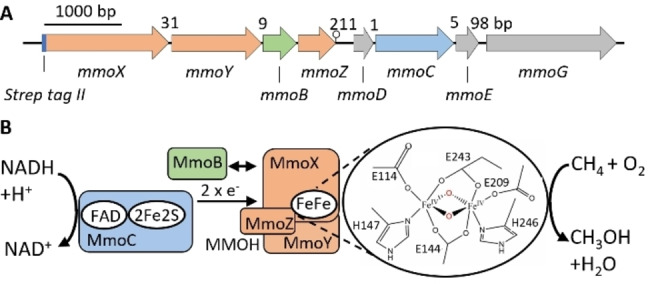
The soluble methane monooxygenase from *M. methanica* MC09. A: Schematic genetic map of the sMMO from *Methylomonas methanica* MC09. Numbers show base pairs between genes. A lollipop symbol between *mmoZ* and *mmoD* indicates a potential terminator. B: Cofactor composition and catalysed reaction of its three components. The enlargement shows the key species Q for methane conversion.^[6]^

Methane is a potent greenhouse gas that is produced mainly by microbial methanogenesis from organic material and anaerobic decomposition of fossil fuels.^[1]^ The biological conversion of green methane into valuable bioproducts such as methanol, polyhydroxyalkanoates, and single‐cell protein has sparked interest as the most reduced form of carbon and the sustainable supply via biogas plants.^[2]^ The NADH‐dependent conversion of methane with the co‐substrate molecular oxygen to methanol is catalysed by soluble methane monooxygenase (sMMO). The sMMO is composed of three components: a NADH‐dependent reductase (MmoC), a regulatory protein (MmoB), and the catalytically active hydroxylase (MMOH) (Figure 1B).[^3^, ^4^]

The MMOH is a (MmoXYZ)_2_ homodimer with the MmoX subunit containing the non‐heme diiron centre, which is coordinated by four glutamates, two histidines, and two bridging hydroxyl molecules between the two irons in the oxidised state.[^3^, ^5^] In contrast to other monooxygenases with a mononuclear Fe(IV)=O intermediates that break weaker C−H bonds, the key reaction cycle intermediate Q with a Fe(IV)_2_O_2_ oxo core (Figure 1B) is capable of activating the inert C−H bond of methane (104 kcal mol^−1^).^[6]^ In addition to methane conversion, sMMOs show high substrate promiscuity and can hydroxylate a variety of molecules, including halogenated aliphatic compounds and aromatic compounds such as naphthalene, which products are attractive for fine chemicals synthesis.^[7]^

MmoC, the sMMO‐specific reductase with a flavin adenine dinucleotide cofactor and a [2Fe−2S] cluster, transfers electrons from NADH to the active site of MMOH (Figure 1B).[^8^, ^9^, ^10^] MmoB, the regulatory component, lacks a prosthetic group. It is necessary for full sMMO activity by modulating the access of gases (CH_4_ and O_2_) to the active centre of MMOH by successively binding after MmoC.[^11^, ^12^, ^13^]

The sMMO operon contains additional open reading frames (designated *mmoD, mmoG* and in some species *orf1/mmoE*) that are unique for diiron enzymes and whose functions are still unknown (Figure 1A). It has been suggested that MmoD and MmoG play a role in MMOH active site assembly, activity, and protein folding.[^12^, ^14^, ^15^]

Many genomes of methanotrophs have been sequenced in recent years, and many uncharacterized sMMOs have been identified in γ‐proteobacteria and α‐proteobacteria.^[16]^ In particular, halotolerant sMMOs may have interesting features in terms of activity, reaction conditions and stability for biotechnological applications.

We recently demonstrated that MmoC from the marine methanotroph *Methylomonas methanica* MC09 is capable of NADH oxidation even at high salt concentrations of up to 2 M.^[8]^ While several MmoC and MmoB have been produced in *Escherichia coli*, the lack of a heterologous system for the MMOH components limits the in‐depth elucidation of the reaction mechanism, protein engineering, and biotechnological applications e. g. biological transformation of gaseous methane to liquid fuels (Bio‐GTL).[^4^, ^17^]

So far, only genetic tools were developed for certain methanotrophs.[^5^, ^18^] Recently, a comprehensive review of the recent development of synthetic methylotrophs was published, in which the authors discussed all the molecular genetic tools developed in this field.^[19]^ Jahng and Wood reported the heterologous production of the sMMO from *Methylosinus trichosporium* OB3b in a *Pseudomonas putida* species. However, the sMMO was exclusively active in trichloroethylene decomposition in *P. putida* F1, and no direct methane hydroxylation was shown.^[20]^ Lidstrom's group developed genetic tools for the industrially relevant γ‐proteobacterium *Methylomicrobium buryatense*, which grows quickly to high cell densities.^[18]^ In this context, the groups of Murrell and Smith achieved homologous sMMO production in *M. trichosporium* OB3b, which is based on homologous recombination, and allowed the alteration of substrate selectivity of sMMO.^[5]^ However, genetic manipulation via conjugation and homologous recombination is time‐consuming, and genetic tools that have so far been developed are limited to few methanotrophs. A plasmid‐based heterologous sMMO production system in *E. coli* would enable simple genetic modifications and affinity chromatography‐based purification, advancing the field of methane‐based biocatalysis significantly. Despite considerable effort from several groups, sMMOs have yet to be produced in an active form in *E. coli*.[^4^, ^17^, ^21^] In this study, we provide experimental evidence that sMMO hydroxylase from the marine methanotroph *M. methanica* MC09 requires the heat shock chaperonin GroES/EL for heterologous production in *E. coli*.

## Results and Discussion

**Figure 2:**
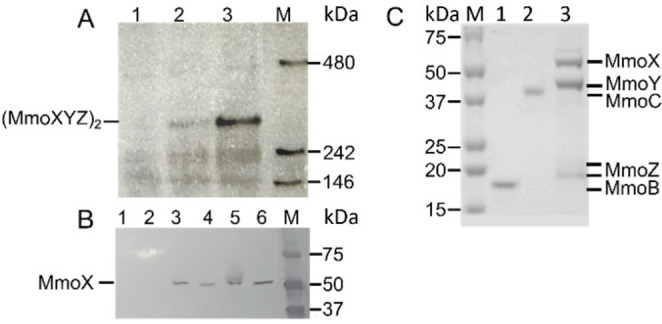
Heterologous produced sMMO in *E. coli*. A: Evaluation of MMOH production by native gel and subsequent immunoblot. Soluble extract protein (20 μg) was added to each lane of a 4–15 % gradient gel. A *Strep*‐AP‐conjugate was used to detect *Strep*‐tagged MmoX. Lane 1: negative control, *E. coli* BL21 without plasmid; lane 2: *E. coli* BL21 with pLL319 (sMMO); lane 3: *E. coli* BL21 with pBB528+pBB541 (GroES/EL)+pLL319 (sMMO); M: native protein marker. Calculated size of [*Strep*‐Tag II–MmoXYZ]_2_ is 252 kDa. B: Evaluation of MmoX production by SDS‐PAGE gel and subsequent immunoblot. 20 μg total protein was added to each lane of a 4–15 % gradient gel. A *Strep*‐AP‐conjugate was used to detect *Strep*‐tagged MmoX. Lanes 1, 3, 5 disrupted cells; lanes 2, 4, 6 soluble extracts; lanes 1, 2 negative control *E. coli* BL21 without plasmid; lanes 3, 4 *E. coli* BL21 with pLL319 (sMMO); lanes 5, 6 *E. coli* BL21 with pBB528+pBB541 (GroEL/ES)+pLL319 (sMMO); M protein marker. Calculated size of *Strep*‐Tag II‐MmoX is 62 kDa. C: Purified sMMO components via affinity chromatography. Coomassie stained SDS‐PAGE 4–20 % gradient gel. M protein marker; lane 1 purified MmoB, lane 2 purified MmoC, lane 3 purified MMOH. 1 μg and 4 μg MmoB/MmoC and MMOH was added to each lane. MMOH was produced in *E. coli* BL21 containing the plasmids pLL319, pBB528, pBB541 and pZD04.

The sMMO gene cluster fragment from *M. methanica* MC09, which contains the *mmoXYBZ* operon, and the *mmoDC*, *orf1/mmoE* and *mmoG* genes (Figure 1A), was cloned into the toluate inducible plasmid pSB‐M1g‐1‐17, and an encoded *N*‐terminal *StrepTag* II was fused to MmoX for detection through immunoblot and subsequent purification, resulting in pLL319 (Figure S3). The initial production of MMOH in *E. coli* at 18 °C in rich TB media supplemented with 54 μM FeCl_2_ resulted in inclusion bodies and only trace amount of (MmoXYZ)_2_ homodimer as shown by immunoblots of SDS‐PAGE and native gels (Figure 2A and 2B). We hypothesized that the chaperonin GroES/EL, whose core component GroEL has a high predicted structural resemblance to MmoG (Figure S4, S5 and S6), would improve protein folding. Indeed, the usage of a co‐synthesizing GroES/EL *E. coli* strain resulted in distinct bands on the SDS‐PAGE/immunoblot belonging to MmoX and on the native gel/immunoblot corresponding to (MmoXYZ)_2_, respectively, indicating accurate subunit assembly of MMOH (Figure 2A and 2B). The requirement of *E. coli* GroES/EL suggests that functional MmoG is not produced in sufficient amounts. This is consistent with the lack of sMMO activity in the soluble extract (not shown), and 211 base pairs between *mmoZ* and *mmoD*, which contains a predicted terminator and a predicted sigma 70‐dependent promotor (Figure 1).[^22^, ^23^, ^24^]

The bacterial chaperonin GroEL forms a 14‐mer porous cylinder (Figure S5 and S6), which is an essential ATP‐driven macromolecular machine for protein folding. The observation of the crucial role of GroES/EL for heterologous MMOH production (Figure 2) coupled with the discovery of MmoG in diverse sMMO operons,^[25]^ together with high structural similarity between MmoG and GroEL (Figure S5 and S6) suggest a similar role of MmoG for protein folding in methanotrophs. While prokaryotic GroEL and GroES form a transient nano‐cage for the substrate protein to fold in isolation (Figure S6), eukaryotic chaperonins contain a “built‐in” lid instead of the GroES module (Figure S7). This “built‐in” lid is missing in the homology model of MmoG (Figures S5, S6 and S7), and the sMMO operons of known methanotrophs lack an encoded “standard” GroES. There are at least three possibilities how MmoG could operate in its native organism: 1^st^: MmoG functions without lid, 2^nd^: the lid is shared with endogenous GroES (encoded 1 Mbp upstream from sMMO operon), or 3^rd^: the hypothetical protein ORF1 (named MmoE) is responsible for closing the folding chamber. Interestingly, the *mmoE* gene is present in sMMO operons of *Methylococcus capsulatus* Bath, *Methylovulum miyakonense* HT12 and *M. methanica* MC09 (Figure S8). However, its sequence similarity to GroES is very low and it bears no structural resemblance to GroEL (Figure S11). Its predicted structure by RoseTTAFold^[26]^ indicates two long twisted alpha‐helices with similarities to a basic leucine zipper domain which is typical of DNA binding proteins (Figure S11). We hypothesized alternative closing of MmoG with the endogenous GroES lid from our docking studies (Figure S09).

Strikingly, chaperonin GroES/EL is required for MMOH folding, which is in line to chaperonin dependent RuBisCO biogenesis. Here the Cpn60/Cpn20, analogous to GroES/EL, support the correct folding of the large RuBisCO subunit, while the specific assembly chaperones RbcX, the RuBisCO accumulation factors 1 and 2, and the Bundle sheath defective‐2 have a supportive role for multimeric RuBisCO assembly.^[27]^ Future crystal structure analysis of MmoG and deletion studies will help elucidate the function and mechanism of the MMOH specific chaperonins.

**Figure 3:**
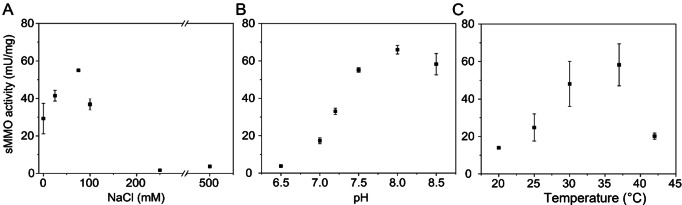
Optimal conditions for the catalysed reaction of soluble methane monooxygenase from *M. methanica* MC09. A: Evaluation of salt tolerance for hydroxylase MMOH with additional MmoZ at optima conditions (37 °C and pH 8.0). B: Evaluation of pH optimum (50 mM NaCl, 37 °C). C: Evaluation of temperature optimum (75 mM NaCl, pH 8.0). The means of three technical replicates and standard deviations are shown.

After demonstrating abundance and multimeric state of MMOH in the soluble cell extract, we used affinity chromatography to purify recombinant MMOH in a single step. The first preparations showed sub‐stoichiometric amounts of the small MmoZ subunit (Figure S12). Co‐synthesising MmoZ on a separate plasmid increased the amount of MmoZ, and conjointly the specific activity by a magnitude (Figure 2C, S13 and S14). We isolated 4–6 mg of pure MMOH per litre *E. coli* cell culture using this four‐plasmid system via *Strep*‐tag affinity chromatography (Figure 2C). For evaluating activity, the reductase MmoC and the regulatory protein MmoB were separately produced in *E. coli* and purified via His_10x_‐tag‐based Ni‐NTA affinity chromatography (Figure 2C).^[8]^ At optimal conditions, the recombinant sMMO system (purified MMOH, MmoB and MmoC from *M. methanica*) showed high specific activity with the well‐established model substrate nitrobenzene (71±1 mU/mg, Figure 3), which is comparable to the activity of native sMMO (150 mU/mg) from *M. trichosporium* OB3b.^[28]^ This demonstrates the successful heterologous production of active sMMO in *E. coli*.

The sMMO exhibited specific activity for only one minute indicating enzyme inactivation (Figure S15). In the presence of catalase, the period of substrate conversion was extended to 10 min with the same calculated slope and thus same turnover rate (Figure S15), providing further evidence that the sMMO reductase MmoC produces hydrogen peroxide.[^8^, ^29^] These findings are comparable to the results shown for sMMO of *M. trichosporium*.^[28]^

Since the MMOH originates from a halotolerant organism, we studied the MMOH at different salt concentrations and determined the optimal reaction conditions. While the isolated sMMO reductase showed significant activity up to 2 M NaCl, the purified MMOH had its optimum activity at 75 mM NaCl and lost it above 200 mM NaCl (Figure 3A) corroborating the hypothesis that the high salt concentration affects the successive binding of MmoB and MmoC. The temperature optimum of 37 °C and the pH optimum of 7.5–8.5 (Figure 3B and 3C) corresponds to the optimal conditions reported for the associated reductase MmoC.^[8]^

Metal determination by inductively coupled plasma optical emission spectrometry (ICP‐OES) and subsequent spectroscopic analysis by UV/Vis and electron paramagnetic resonance spectroscopy (EPR) provided more information on correct diiron active site insertion. Based on ICP‐OES measurements the iron content of the isolated MMOH was estimated to be 45±0.18 %. Reconstitution of purified MMOH with iron as described in[^3^, ^30^] had no effect on the specific activity (Figure S16). The UV/Vis spectrum of as isolated MMOH showed a high protein absorbance at 280 nm (Figure S17), but no further absorption features could be observed over 300 nm. These results are in line to previous observations.^[31]^ Structure prediction with SWISS‐MODEL^[32]^ indicates coordination of the diiron active site via the conserved E114, E144, H147, E209, E243, H246 residues (Figure 1B, S18 and S19). The EPR spectrum of as‐isolated sMMO exhibits only a weak rhombic signal (g_x_=1.95, g_y_=1.87, g_z_=1.77, see Figure S22). However, this spectral feature is characteristic for the mixed‐valence state of the diiron site in MMOH. The g‐values are similar to well‐characterized MMOHs from other organisms and indicate a native active site incorporation and electronic structure.[^3^, ^33^] In line with the oxidative conditions during protein purification, the largest part of the enzyme presumably remains in the di‐ferric state.

In conclusion, we provide evidence that GroES/EL is required for desired sMMO hydroxylase folding by successfully producing in *E. coli*. A recent preprint about a synthetic *E. coli* strain with sMMO activity after co‐synthesis of GroESL supports our study.^[34]^ Heterologous production in *E. coli* allowed rapid purification at mild conditions of all three recombinant sMMO components via affinity chromatography. The correct subunit assembly of active sMMO hydroxylase and insertion of its diiron active site was demonstrated by a set of biochemical and spectroscopic techniques. The sMMO from the marine *M. methanica* MC09 represents a promising biocatalyst for hydroxylation reactions under a wide range of reaction conditions including moderate salt concentrations.

The heterologous production of sMMO allows the elucidation of details of the sMMO reaction mechanism through site directed mutagenesis and its biosynthesis via deletion studies. The use of a platform for heterologous sMMO simplifies the application of sMMO in biotechnological approaches. Examples are gas to liquid processes, the use of methane as a C1 carbon source, and the synthesis of hydroxylated fine chemicals.

## Conflict of interest

The authors declare no conflict of interest.

1

## Supporting information

As a service to our authors and readers, this journal provides supporting information supplied by the authors. Such materials are peer reviewed and may be re‐organized for online delivery, but are not copy‐edited or typeset. Technical support issues arising from supporting information (other than missing files) should be addressed to the authors.

Supporting Information

## Data Availability

The data that support the findings of this study are available in the supplementary material of this article.
