# Supplementary material for: Crucial Role of the Chaperonin GroES/EL for Heterologous Production of the Soluble Methane Monooxygenase from Methylomonas methanica MC09
Source: Chembiochem. 2022 Apr 29;23(12):e202200195. doi: 10.1002/cbic.202200195 (PMC9324122; doi:10.1002/cbic.202200195)
Supplement: Supplementary file 1 — Supporting Information [file CBIC-23-0-s001.pdf]

ChemBioChem

Supporting Information

Crucial Role of the Chaperonin GroES/EL for Heterologous Production of the Soluble Methane Monooxygenase from *Methylomonas methanica* MC09

Domenic Zill, Elisabeth Lettau, Christian Lorent, Franziska Seifert, Praveen K. Singh, and Lars Lauterbach*

Author Contributions

D.Z. Data curation:Lead; Formal analysis:Lead; Methodology:Lead
E.L. Methodology:Supporting; Supervision:Supporting; Validation:Supporting; Visualization:Supporting; Writing – review & editing:Supporting
C.L. Data curation:Supporting; Formal analysis:Supporting; Validation:Supporting
F.S. Methodology:Supporting; Validation:Supporting; Writing – review & editing:Supporting
P.S. Project administration:Supporting; Validation:Supporting; Visualization:Supporting; Writing – review & editing:-Supporting
L.L. Conceptualization:Lead; Data curation:Equal; Funding acquisition:Lead; Methodology:Supporting; Supervision:Equal; Visualization:Equal; Writing – original draft:Lead; Writing – review & editing:Equal

Plasmid construction

Plasmids used in this study are listed in Table S1, shown as plasmids maps in Figure S1-4 and their sequences are presented at the end of this SI in Figure S18-22. The expression plasmid used for overproduction of the Strep-tagged sMMO hydroxylase from *Methylomonas methanica* MC09 was constructed as follows. Plasmid pSB_M1g-1-17with an encoded GFP under control of the toluate inducible *xyl*S/Pm promotor variant ML1-17 served as the basis for the subsequent cloning steps. [1] The *gfp* gene was equipped at the 5′ end with a Strep-tag II-encoding sequence via NEBuilder assembly and the oligo 207_StreptagII (see Table S2). From the resulting plasmid pLL210, the *Spe*I restriction digested 7.8 kb fragment was assembled with the 7.3 kb fragment mmoXYBZDC_Orf1_mmoG fragment from *M. methanica*, which was amplified with the primers 255_Mm and 256_Mm (Table S2). This step yielded plasmid pLL319, which was transferred via electroporation into *E. coli* BL21.

For heterologous overproduction and subsequent purification, the *mmoB* gene from *Methylomonas methanica* MC09 was codon optimised for *E. coli* and inserted via the *Nde*I and *Bam*HI restriction sites in the plasmid pET-16b. The 5´ end of *mmoB* was equipped with a 10x His-tag-encoding sequence resulting in the plasmid pZD01 (Figure S1). For co-synthesis of MmoZ, the mmoZ gene from *M. methanica* MC09 was codon optimised for *E. coli* and inserted via the *Sal*I and *BamH*I restriction sites in the plasmid pETDuet-1. The 5´ end of *mmoZ* was equipped with a 6x His-tag-encoding sequence resulting in the plasmid pZD04 (Figure S2).

Table S1: Plasmids used in this study

Name	Key feature	Source
pSB_M1g-1-17	*xylS/Pm* promotor variant ML1-17, RK2 replicon, *gfpmut3,* Kan^R	Bakke *et al.* 2009[2]; Balzer *et al.* 2013[1]
pLL210	pSB_M1g-1-17 with an encoded *Strep-tag II* in front of *gfpmut3,* Kan^R	This study
pLL319	7.3 kb *mmoXYBZDC*_Orf1_*mmoG fragment from M. methanica* in pLL210 with *mmoX eq*uipped at 5´ with an encoded *Strep-tag II,* Kan^R	This study
pZD01	*mmoB* from *M. methanica* MC09 in pET-16b, Amp^R	This study
pZD02	*mmoC* from *M. methanica* MC09 in pET-16b	Lettau *et al.* 2021[3]
pZD04	*mmoZ* from *M. methanica* MC09 in pETDuet, Amp^R	This study
pBB528 + pBB541	groES/EL Spec^R, Chlor^R	De Marco 2007[4]

Table S1: Oligos used in this study

Oligo	sequence
207_StreptagII	CTTTATCAACATGTACAATAATAATGGAGTCATGAACATATGGCTAGCTGGAGCCACCCGCAGTTCGAAAAAGGCGCCACTAGTTTAATTAATGCGGTACCAAGTAAAGGAGAAGAAC
255_Mm	CCCGCAGTTCGAAAAAGGCGCCACTATGGCTATAAGTGCAGCAAC
256_Mm	CTTTACTTGGTACCGCATTAATTAAATCTCTCGATTTTGACTTGCC

sMMO production and purification

E. coli BL21 with the plasmids pLL319 + pBB528 + pBB541 for the co-production of the chaperonin GroES/EL and additional pZD04 (MmoZ) were grown in rich Terrific Broth (TB) medium at 37 °C until OD_{600nm} of 2 and were then induced with 2 mM toluate and 1 mM IPTG. The protein production phase was performed at 18 °C for 30 h. The harvested cells were resuspended in twice their volume of resuspension buffer (500 mM NaCl, 50 mM KPO_4, pH 7.2 containing additional Protease Inhibitor (EDTA-free, Roche) and DNase I). After two passages through a chilled French press at a pressure of 6.2 MPa, the suspension was centrifuged at 100,000x g for 45 min. The soluble extract was applied to a 2 mL Strep-tag Superflow affinity chromatography column, washed with 6 mL of resuspension buffer with protease inhibitor and eluted with 12 mL of 5 mM desthiobiotin solution. The eluate was then concentrated in an Amicon Ultra-15 centrifugal cell (100K membrane; Amicon, Witten, Germany). To remove desthiobiotin after Strep-tag Superflow affinity chromatography, the elution buffer of MMOH was exchanged via an illustra NAP-10 column (GE Healthcare UK Limited, Buckinghamshire, UK) to 25 mM MOPS, 100 mM NaCl, 0.2 mM $(NH_4)_2Fe(SO_4)_2$, 1 mM TCEP, pH 7.2. Protein concentration was determined with BCA protein assay kit (Pierce, USA) as described previously.[5]

We isolated about 0.83 ± 0.63 mg (average of total five purifications) homogenous MMOH from 1 g cell pellet (wet weight). In order to monitor the purification, samples of every purification step were analysed by SDS-PAGE (Figure S13). In the elution fraction, the pronounced bands were detected, which correspond to the three MMOH subunits calculated size of Strep-tagged-MmoX 62.0 kDa, MmoY 45.1 kDa, and MmoZ 18.9 kDa. A weak second band above the MmoZ band indicates the 6xHis-tagged MmoZ derivative (calculated size 20.5 kDa). Because of the already high purity of MMOH (> 95%), we decided to not proceed with further purification steps.

Spectroscopic measurements

Samples UV/visible spectra were recorded with a Varian Cary 60 (Agilent Technologies) at 20°C. The final working concentration of protein samples was around 45 mg/ml. Measurements were performed in 25 mM MOPS buffer with 100 mM NaCl at pH 7.2.

Electron Paramagnetic Resonance (EPR) spectroscopy was conducted on a Brucker EMX plus X-Band spectrometer equipped with an ER 4122 super-high Q resonator and an Oxford ESR900 helium flow cryostat. An Oxford ITC4 temperature controller was used for adjusting the temperature. The baseline was corrected by subtracting a reference spectrum of buffer solution recorded with the same experimental parameters. For subsequent corrections a spline function was used. Experimental parameters used were: microwave power 1 mW, microwave frequency 9.29 GHz, modulation amplitude 10 G, and modulation frequency 100 kHz. The as isolated MMOH was analyzed at a concentration of 55 µM and a volume of 100 µL.

Optical emission spectroscopy

For the determination of iron in MMOH, metal analysis was performed using a Perkin-Elmer Optima 2100DV inductively coupled plasma-optical emission spectrometer (Perkin-Elmer, Fremont, CA, USA) following the protocol described previously[6]. In short, 500 µL of protein samples were incubated overnight with equal amount of 65% nitric acid (Suprapur, Merck KGaA, Darmstadt, Germany) at 100 °C. Samples were filled up to 5 mL with water prior to ICP-OES analysis. Buffer samples without protein were treated the same way to check if footprint of metal is dissolved in the buffer. As reference, the multielement standard solution XVI (Merck) was used.

Activity measurements

The hydroxylation activity of MMOH was measured with nitrobenzene as the substrate under aerobic conditions (75 mM NaCl, 25 mM MOPS buffer, pH 8.0, 1 mM nitrobenzene). Proteins MMOH, MmoB and MmoC were added in the ratio H:B:C = 2:2:1. The reaction was started by adding 0.5 mM NADH. The production of p-nitrophenol was followed at 420 nm (VARIAN Cary 50 BIO UV-Visible Spectrometer, $\varepsilon = 11.861\ mM^{-1}\ cm^{-1}$ at pH 8.0). The production of p-nitrophenol is followed at 405 nm due to interferences with NADH at shorter wavelength.[7] For the determination of reaction optima, first the optimum for salt concentration, and then the pH optimum were determined at 37 °C. Finally, the determination of the temperature optimum was performed. Salt concentrations were analysed in the range of 0-0.5 M NaCl (due to marine habitat from *M. methylomonas*), pH values ranged from 6.5-8.5, and temperature studies were performed from 10-42 °C. Furthermore, activity assays with addition of different catalase concentrations (0-8550 U/mg) were performed, because of the possibility of H_2O_2 production by MmoC, which could inhibit the sMMO activity. For the determination of optima conditions, no catalase was added. Each measurement was performed with technical triplicates but also with biological replicates.

For the exact calculation of the activity, the extinction coefficient of p-nitrophenol was determined additionally. Absorption of p-nitrophenol (0.1 mM in 25 mM MOPS buffer with 75 mM NaCl) was measured at different pH at 420 nm with three technical replicates. Law of Lambert-Beer was used to calculate the extinction coefficient. The extinction coefficients for pH 7.2 (used for purification) and pH 8.0 (used for activity measurements) were 9.164 and 11.861 $mM^{-1}cm^{-1}$, respectively.

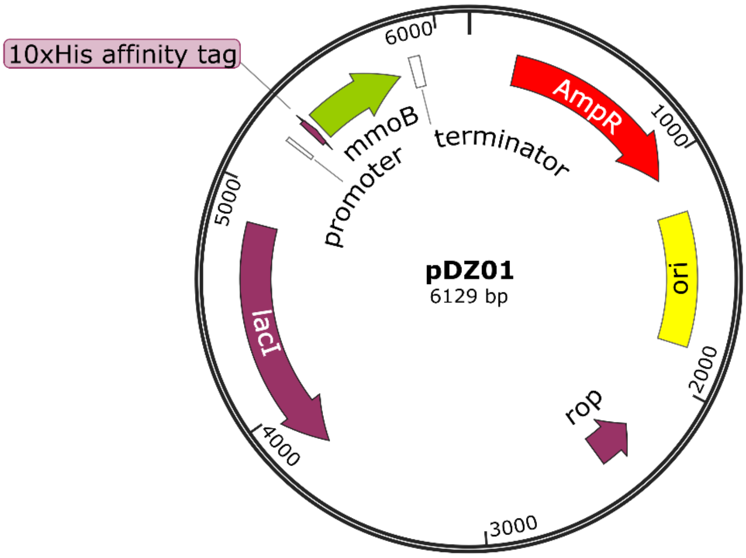

Figure S1: Plasmid map of pZD01 used for overproduction of MmoB from *M. methanica* MC09 (generated with SnapGene software, GSL Biotech, USA).

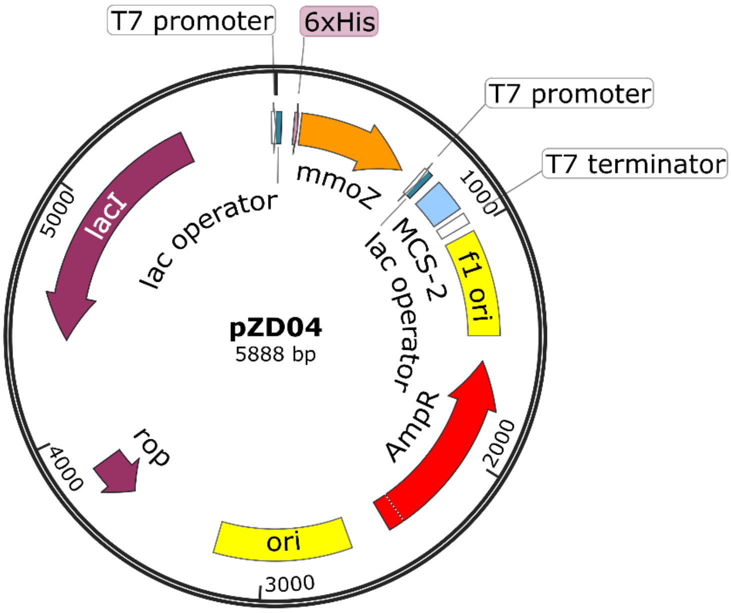

Figure S2: Plasmid map of pZD04 used for overproduction of MmoZ from *M. methanica* MC09 (generated with SnapGene software, GSL Biotech, USA).

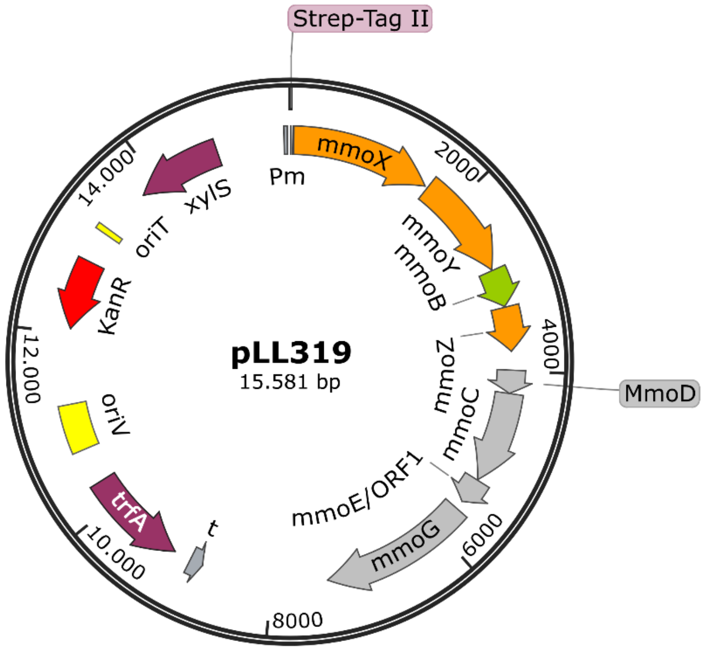

Figure S3: Plasmid map of pLL319 used for overproduction of the sMMO hydroxylase from *M. methanica* MC09 (generated with SnapGene software, GSL Biotech, USA).

```
EcK12GroEL         --------------------MAAKDVKFGNDARVKMLRGVNVLADAVKVTLGPKGRNVVL        40
PpF1GroEL          --------------------MAAKDVKFGDSARKKMLVGVNVLADAVKATLGPKGRNVVL        40
CnH16GroEL         --------------------MAAKDVVFGDAARAKMVEGVNILANAVKVTLGPKGRNVVL        40
TtHB8GroEL         ---------------------MAKILVFDEAARRALERGVNAVANAVKVTLGPRGRNVVL        39
XoGroEL            --------------------MAAKDIRFGEDARTRMVRGVNVLANAVKATLGPKGRNVVL        40
McBathMmoG         ---------------------MAKEVVYRGSARQRMMQGIEILARAAIPTLGATGPSVMI        39
MjNIMmoG           ---------------------MTIDVIFNPEASERVLSGIRTVARAASVTFGSSGPSVVI        39
MtOB3bMmoG         MTNPRKRERRRPAFDVTREKFVARNIRFGDVVRRDLLAGVDALADAVAVTLGPRGRNVVI        60
MsiBL2MmoG         ---------------------MAKDIRYGDAARRRMLAGANLLADAVQVTLGPRGRNVVI        39
MspMmoG            ------------------------------------------------------------        0
MmiHT12MmoG        ---------------------MSKEIIYNPEIRVRLMAGINQVARAVAVTYGRVGSTVMI        39
MmMC09MmoG         ---------------------MSKQVIYNPEARQRLLQGINAVARAAGVTLGSAGPAVMI        39

EcK12GroEL         DKSFG--APTITKDGVSVAREIELEDKFENMGAQMVKEVASKANDAAGDGTTTATVLAQA        98
PpF1GroEL          AKSFG--APTITKDGVSVAKEIELKDAFENMGAQLVKEVASKANDAAGDGTTTATVLAQA        98
CnH16GroEL         ERSFG--GPTVTKDGVSVAKEIELKDKLQNMGAQMVKEVASKTSDNAGDGTTTATVLAQS        98
TtHB8GroEL         EKKFG--SPTITKDGVTVAKEVELEDHLENIGAQLLKEVASKTNDVAGDGTTTATVLAQA        97
XoGroEL            EKSFG--APTITKDGVSVAKEIELADKFENMGAQMVKEVASKTNDNAGDGTTTATVLAQA        98
McBathMmoG         QHRADGLPPISTRDGVTVANSIVLKDRVANLGARLLRDVAGTMSREAGDGTTTAIVLARH        99
MjNIMmoG           QHRTDGIPPIITRDGVTVAKSIQFEDRVADLGARMLRDVAGSVSREVGDGTTTAIVLAQT        99
MtOB3bMmoG         EHRAAGLPPVATKDGVTVAQAVELAGRTQSVGVSLVRQMATAVAKEAGDGTTTSVVLARR        120
MsiBL2MmoG         QHRTSGILPVVTKDGVTVARSIAVDDRFESAGINMFKEMAGRVSKECGDGTTTTIVLARF        99
MspMmoG            ---------------------------MESVGITLVRQMATTVAKEAGDGSTTSVVLTHR        33
MmiHT12MmoG        QHRTDGIMPVFTRDGVTVANAVLIQDRIADLGGRMLRDVAGAMSRQVGDGTTTAIVLAQV        99
MmMC09MmoG         QHRTEGIMPIFTRDGVTVANAIVMEDRIADLGARMLRDVAGSVSREVGDGTTTAIVLAQS        99
                                                .   *  :.:::*       ***:**: **::

EcK12GroEL         IITEGLKAVAAGMNPMDLKRGIDKAVTAAVEELKALSVPCSDSKAIAQVGTISANSDETV        158
PpF1GroEL          IVNEGLKAVAAGMNPMDLKRGIDKATAAVVAELKNLSKPCADSKAIAQVGTISANSDNSI        158
CnH16GroEL         IVREGMKFVAAGMNPMDLKRGIDKAVAAAVEELKKVSKPTTTSKEIAQVGAISANSDTSI        158
TtHB8GroEL         IVREGLKNVAAGANPLALKRGIEKAVEAAVEKIKALAIPVEDRKAIEEVATISAN-DPEV        156
XoGroEL            LIREGAKAVAAGMNPMDLKRGIDQAVKAAVVELKNISKPTTDDKAIAQVGTISANSDESI        158
McBathMmoG         IAREMFKSLAVGADPIALKRGIDRAVARVSEDIGARAWRGDKESVILGVAAVATKGEPGV        159
MjNIMmoG           LAIESIKSVAAGFHPLQIKQGLEGALAIVEAQLQSMALIYSGLDWLESLAMVATKQEQAA        159
MtOB3bMmoG         LAAETRKALAAGMNPRDIVLGMEKAARIVDRDLAARARRCDDTRALAHVATLAAGGDESI        180
MsiBL2MmoG         IARKVLRAMSSGLDPNGLRTGLELATQTAVEDLKRRAKSCADERSIVHIAATASNGDLSV        159
MspMmoG            VAAETRKALAAGMNPRDVSLGMERAARAVEADLLRRSRRCDDRRSLAHVATLAAGGDEGI        93
MmiHT12MmoG        LAQGCLKSVAAGFHPMQLKKGLDVALAVVEKNLLERAVTGVTSDWVEKIALVASKNETKA        159
MmMC09MmoG         LAADALKSVAAGFHPLQLRKGMELALSLVEQHLKKTALTGVTEDWVEKIALVATKGEAGV        159
                   :     : :: * .*  :  *:: *   .  .:   :        :  :.  ::  :

EcK12GroEL         GKLIAEAMDKVGKEGVITVEDGTGLQDELDVVEGMQFDRGYLSPYFINKPETGAVELESP        218
PpF1GroEL          GEIIAEAMEKVGKEGVITVEEGSGLENELSVVEGMQFDRGYLSPYFVNKPDTMVAELESP        218
CnH16GroEL         GERIAEAMDKVGKEGVITVEDGKSLADELEVVEGMQFDRGYLSPYFINNPEKQVVQLDNP        218
TtHB8GroEL         GKLIADAMEKVGKEGIITVEESKSLETELKFVEGYQFDKGYISPYFVTNPETMEAVLEDA        216
XoGroEL            GNIIAEAMKKVGKEGVITVEEGSGLENELDVVEGMQFDRGYLSPYFINNQQSQSADLDDP        218
McBathMmoG         GRLLLEALDAVGVHGAVSIELGQRREDLLDVVDGYRWEKGYLSPYFVTDRARELAELEDV        219
MjNIMmoG           SRLLAKAHQELDGKGELSFELGNSREDELEIVDGLRYEQGYLSPYFVTDKDRAEAVLDNP        219
MtOB3bMmoG         GAIVADALTRAGEGGVVDVELGAALCDEMDIVEGMRWEQGYRSPYFMTDSARKIAELENP        240
MsiBL2MmoG         GELLASAFKKVGPNGIVNVSLGNGTSDEIAFQEGAHWEQGWLSPYFMTDKTRRIAELINP        219
MspMmoG            GEIVAEALEIAGDGGVVDVELGNGVTDEIESVEGMHWEQGYRSPYFMTDSARKIAELENP        153
MmiHT12MmoG        GKLLAQAFDELGLQRELTFLLGNGLADELAIVDGVQYGQGYLSPYFITDKTRAEAVLENP        219
MmMC09MmoG         GKLLSRALSELGDDGSLTFQLGNGREDQLEVVEGIHYQQGFLSPYFITDKTRGEAVLEQP        219
                   .  :  *    .    : .  .      :   :* :: :*: ****:..     . * .

EcK12GroEL         FILLADKKISNIREMLPVLEAVAKAGKPLLIIAEDVEGEALATLVVNTMRGIVKVAAVKA        278
PpF1GroEL          LLLLVDKKISNIRELLPVLEAVAKAGRPLLIVAEDVEGEALATLVVNNMRGIVKVAAVKA        278
CnH16GroEL         FVLLFDKKISNIRDLLPVLEQVAKAGRPLLIVAEDVEGEALATLVVNNIRGILKTAAVKA        278
TtHB8GroEL         FILIVEKKVSNVRELLPILEQVAQTGKPLLIIAEDVEGEALATLVVNKLRGTLSVAAVKA        276
XoGroEL            FILLHDKKISNVRDLLPVLEGVAKAGKPLLIVAEEVEGEALATLVVNTIRGIVKVVAVKA        278
McBathMmoG         YLLMTDREVVDFIDLVPLLEAVTEAGGSLLIAADRVHEKALAGLLLNHVRGVFKAVAVTA        279
MjNIMmoG           YILLYDREIGDLMDLIPILEQVREQDRSLLIIAENVIDKALTGLLLNHVRGVFRAVAVKP        279
MtOB3bMmoG         YILIYDRVINQFSELVPALELVRRQRGSLLIVAENIVEEALPGLLLNHIRKNLCSIAVKG        300
MsiBL2MmoG         YVLLYDRPIKQFDELIPILDQVRQAEGSLLIIADDIEEAALGGILLNHIRCVLKAVAVKP        279
MspMmoG            YILIYDRVINEFSELVPALELVRRRGGSLLVVAENIAEEALPGLLLNHIRKNLCSIAVKG        213
MmiHT12MmoG        YILFYDREINDLMELVPILEQVKAQGRPLLVVAEDVVDKALTGLLLNHIRGIFKVVAVKP        279
MmMC09MmoG         YILLYDREIDDFIDLVPILEEVAAEGRPLLLIAESVSEKALAGLLLNHVRGNFKVVAVKP        279
                    :*: :: : :. :::* *: *      **: *: :   **  :::* :*  .   **.

EcK12GroEL         PGFGDRRKAMLQDIATLTGGTVISEEIGM--ELEKATLEDLGQAKRVVINKDTTTIIDGV        336
PpF1GroEL          PGFGDRRKAMLQDIAVLTGGQVISEEIGL--TLETTTLEHLGNAKRVILSKENTTIIDGA        336
CnH16GroEL         PGFGDRRKAMLEDIAILTGGTVIAEEIGL--TLEKAGLNDLGQAKRIEIGKENTIIIDGA        336
TtHB8GroEL         PGFGDRRKEMLKDIAAVTGGTVISEELGF--KLENATLSMLGRAERVRITKDETTIVGGK        334
XoGroEL            PGFGDRRKAMLEDMAVLTGGTVISEEVGL--ALEKATIKDLGRAKKVQVSKENTTIIDGA        336
McBathMmoG         PGFGDKRPNRLLDLAALTGGRAVLEAQGD--RLDRVTLADLGRVRRAVVSADDTALLGIP        337
MjNIMmoG           PGFGDRRRDRLNDLAVLTGGQAILEDGLL--TLDTIDLSHLGQARRVIINEFTTTIIGAS        337
MtOB3bMmoG         PGYGDSRYEFLHDLAALTGGRAIMEACGE--ELSNVTMAHLGRAKRVVVREDDTVVIGGE        358
```

```
MsiBL2MmoG        PAYGDRRKETLADLACLLGGRAILEDNGD--ELSHVKLADLGRANRAEVTESETTLFGGA      337
MspMmoG           PGYGDSRYEYLLDLAALTGGRAIMEAFGE--DISNVTIEHLGRARRVVVREDDTLVIGGE      271
MmiHT12MmoG       PGFGDQRINRLKDLALLTGGQAILDGHGLSLRLEHVGLEQLGQAQRAVITESSTTIIGAV      339
MmMC09MmoG        PGFGDARINRLSDLALLTGGTAILEAHAP--RLEQVKLTQLGQAQRAVINEGSTTVIGAA      337
                  *.:** *   * *:* : ** .: :       :.   :  **...:  :    * :..

EcK12GroEL        GEEAAIQGRVAQIRQQIEEAT-----------SDYDREKLQERVAKLAGGVAVIKVGAAT      385
PpF1GroEL         GVDADIEARVKQIRAQIEETS-----------SDYDREKLQERLAKLAGGVAVIKVGAGT      385
CnH16GroEL        GDAAAIEGRVKQIRAQIEEAT-----------SDYDREKLQERVAKLAGGVAVIKVGAAT      385
TtHB8GroEL        GKKEDIEARINGIKKELETTD-----------SEYAREKLQERLAKLAGGVAVIRVGAAT      383
XoGroEL           GDSAAIESRVGQIKTQIEDTS-----------SDYDREKLQERVAKLAGGVAVIKVGAST      385
McBathMmoG        GTEAS-RARLEGLRLEAEQYRALKPGQGSATGRLHELEEIEARIVGLSGKSAVYRVGGVT      396
MjNIMmoG          GDPEQIEPLIARLSREADLVRARRPGEGSPTGNMHELEELEERINALSGKNGIFKVGGST      397
MtOB3bMmoG        GDGAAITERLAAARQQADWITDGDPSKGSPSGKRHDLENLQTRIKALSGKVVTIKAGGLS      418
MsiBL2MmoG        GDADKIAERVHALRFEAERLQK--NDRGSPTGKLHDLEEFDERIGNLSSVTATIHVGGGT      395
MspMmoG           GDAAVIADRLASAKRQADWIVEGDGSKGSPSGKRHELQNLQTRIKALSGRMATIRAGGLS      331
MmiHT12MmoG       GDPAAIAQLSETLRQEAGLILAKKPGAGSATGNKHDFDELQDRLALLSGKTGTFSVGGNT      399
MmMC09MmoG        GAGEPILERIGALRRQLAAVNARKPGAGSPSGNLHEAEELEERIAVLSGKTGAYSVGGTT      397
                  *              :                  :  :::: *:  *:.      .*. :

EcK12GroEL        EVEMKEKKARVEDALHATRAAVEEGVVAGGGVALIRVASKLADLRGQ-NEDQNVGIKVAL      444
PpF1GroEL         EVEMKEKKARVEDALHATRAAVEEGVVPGGGVALVRALAAIVDLKGD-NEDQNVGIALLR      444
CnH16GroEL        EVEMKEKKARVEDALHATRAAVEEGIVPGGGVALLRARAAISALTGE-NADQNAGIKIVL      444
TtHB8GroEL        ETELKEKKHRFEDALNATRAAVEEGIVPGGGVTLLRAISAVEELIKKLEGDEATGAKIVR      443
XoGroEL           EIEMKEKKARVEDALHATRAAVEEGVVPGGGVALVRALVAVGNLTGA-NEDQTHGIQIAL      444
McBathMmoG        DVEMKERMVRIENAYRSVVSALEEGVLPGGGVGFLGSMPVLAELEAR-DADEARGIGIVR      455
MjNIMmoG          DFEIKERMVRIENAYKSIQAAMAEGVIPGCGIGLYRCIEALREPIAD-D-ERQHAVRIMQ      455
MtOB3bMmoG        DILIKERMQRIENALASARAARSDGVVAGGGVGLYRARAALTEATGD-TLDQTYGIAIVR      477
MsiBL2MmoG        ETEMKERLQRVENARNAVAAALAEGGLPGGGAGLLRCRKALSGLSST-DIAVRHGIQIIA      454
MspMmoG           DIVIKERMQRIENALNSARSAQNDGVVAGGGVGLYRARAALAELRGD-NLDQDHGVAIVR      390
MmiHT12MmoG       DFEMKERMVRLENAYLSAKAALEEGVLPGGGVALYHCREAIEKAIAE-NVGQQQGFRILQ      458
MmMC09MmoG        DVEIKERLVRIENAYLSAKAAVAEGVLPGGGVGLFRCRILLNEVIAE-NAEQQQGVAIIK      456
                  :  :**:  *.*:*  :  :*  :* : * *  :      :             .  :

EcK12GroEL        RAMEAPLRQIVLNCGEEPSVVANTVKGG--DGNYGYNAATEEYGNMIDMGILDPTKVTRS      502
PpF1GroEL         RAVEAPLRQITANAGDEPSVVADKVKQG--SGNYGYNAATGEYGDMIEMGILDPAKVTRS      502
CnH16GroEL        RAMEEPLRQIVLNAGEEASVVVAKVIEG--KGNYGYNAASGEYGDLVEMGVLDPTKVTRT      502
TtHB8GroEL        RALEEPARQIAENAGYEGSVIVQQILAETKNPRYGFNAATGEFVDMVEAGIVDPAKVTRS      503
XoGroEL           RAMEAPLREIVANAGEEPSVILNKVKEG--TGNYGYNAANGEFGDMVEFGILDPTKVTRS      502
McBathMmoG        SALTEPLRIIGENSGLSGEAVVAKVMDH-ANPGWGYDQESGSFCDLHARGIWDAAKVLRL      514
MjNIMmoG          EALRAPARQLLINAGVNPETVFAVIDSD-RDVNITFDTIQNRFGNYLDIGVVDSVKIVRM      514
MtOB3bMmoG        AALDEPIRRIAANAGRDAHEFLFELKRS-NDDFWGMDMRSGECGDLYAAGVIDPARVTRL      536
MsiBL2MmoG        DAVGEPLRRIADNSGKDALAVAYETLAS-SDEGFGCDARTGRFGDLFEAGVIDPLRVTRL      513
MspMmoG           AALDEPIRRIAANAGVDADEFLFELRRS-NDDFWGMDMRSGACGDLFAAGVIDPVRVTRL      449
MmiHT12MmoG       QALAAPLLTLIQNAGLNNEAVLNQLDTL-GDDHITLDTENHRYGNFLEIGIIDPVKVMRL      517
MmMC09MmoG        NALGAPMRQLLSNGGLNSEEVMMRLCGQ-TDPQFAFDMRHQRYGHFLDIGIIDSVKVVRL      515
                   *:  *   :  * * .   .               :       .    *: *  :: *

EcK12GroEL        ALQYAASVAGLMITTECMVTDLPKNDAADLGAAGGMGGMGGMGGMM  548
PpF1GroEL         ALQAAASIGGLMITTEAMVADLPEDKPA--GGMPDMGGMGGMGGMM  546
CnH16GroEL        ALQNAASVASLMLTTDCAVAESPKEESAP-AMPGGMGGMGGMEGMM  547
TtHB8GroEL        ALQNAASIGALILTTEAVVAEKPEKKESTPA----SAGAGDMDF--  543
XoGroEL           ALQNAASIAGLMITTEAMVADAPKKDEPAMPAGGGMGGMGGMDF--  546
McBathMmoG        ALEKAASVAGTFLTTEAVVLEIPDTDAFAG-FSAEWAAATREDPRV  559
MjNIMmoG          ALRNAVSVITTLITAETVLMHVPDLSIMAG-YSPEWAAATREDPRE  559
MtOB3bMmoG        ALRNAVATASSLMTVECAVTHIPPSDPTYG-FDPHLAAATREDPRS  581
MsiBL2MmoG        ALQHATITASTLMTTECVVANLPPDDPTFG-YTGEWAAATREDPRL  558
MspMmoG           ALRNAVATASSLMTVECAVTHIPPSDPTFG-FDARRAAETREDPRA  494
MmiHT12MmoG       ALRNAVSVVGTLITSETVVMEVPDLSLMAG-YSPEWAAATREDPRV  562
MmMC09MmoG        ALRKAVSVVGTLISSDTVIMNVPDLSIMDG-YSAEWAAATREDPRS  560
                  **. *.     ::: :  : . *  .          ..
```

Figure S4. Multiple Sequence alignment of MmoG with GroEL. Sequences of GroEL from *Escherichia coli* K12 (*Ec*K12), *Pseudomonas putida* F1 (*Pp*F1), *Cupriavidus necator* H16 (*Cn*H16), *Thermus thermophilus* HB8 (*Tt*HB8) and *Xanthomonas oryzae* (*Xo*) and sequences of MmoG from methanotrophs *Methylococcus capsulatus* BATH (*Mc*Bath), *Methylomicrobium japanese* NI (*Mj*NI), *Methylosinus trichosporium* OB3b (*Mt*OB3b), *Methylocella silvestris* BL1 (MsiBL1), *Methylosinus sporium* (*Msp*), *Methylovulum miyakonense* HT12 *(Mmi*Ht12) and MmoE from *Methylomonas methanica* MC09 (*Mm*MC09) were shown.

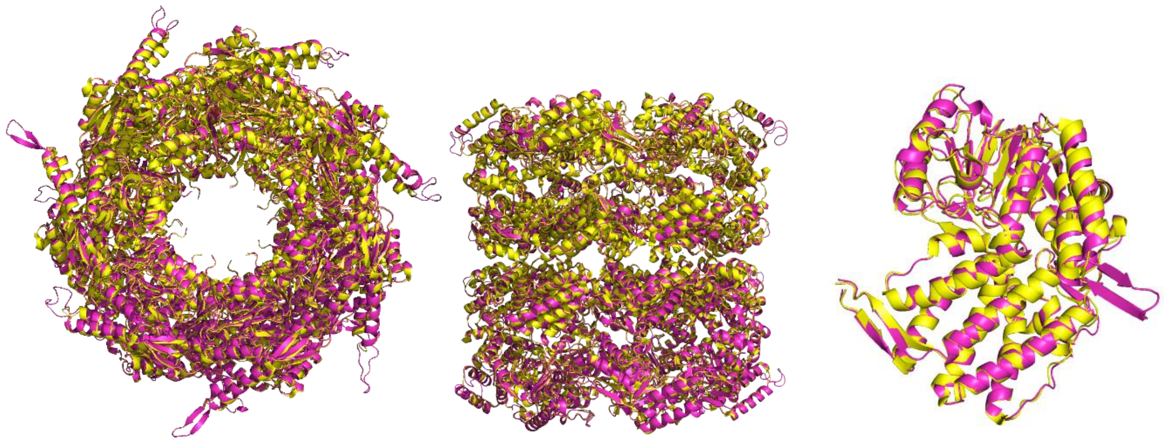

Figure S5: Homology model of MmoG (pink) from *M. methanica* MC09 without lid calculated by using SWISS-MODEL[8] based on PDB: 6KFV (GroEL in yellow, homo-14-mer) with a model quality estimation (QMEANDisCo Global) of 0.70 ± 0.05. Left top view, middle side view, left single subunit.

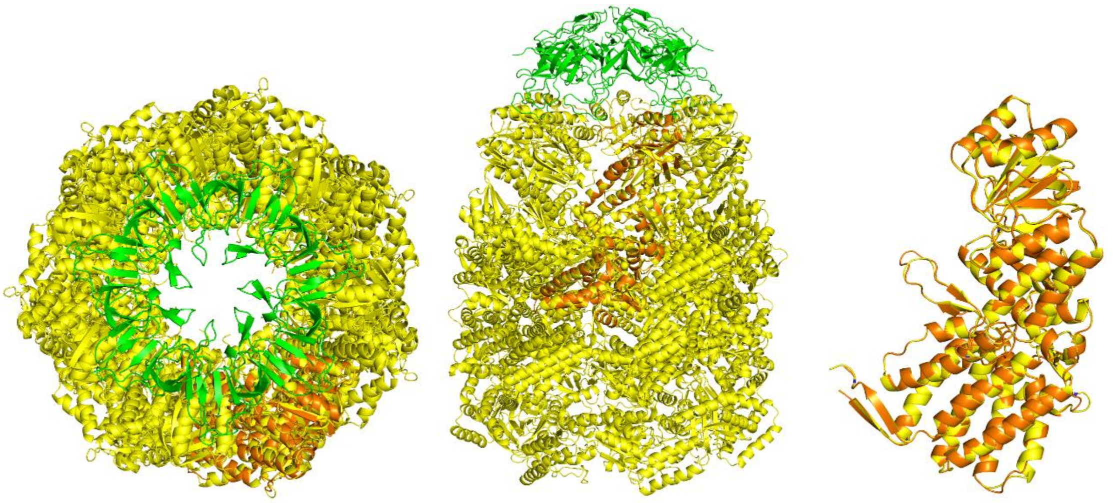

Figure S6: Homology model of MmoG (orange) from *M. methanica* MC09 calculated by using SWISS-MODEL[8] based on PDB: 4V4O (GroEL in yellow and with its “lid” GroES and bound substrate in green) with a model quality estimation (QMEANDisCo Global) of 0.72 ± 0.05. Left top view, middle side view, right single subunit.

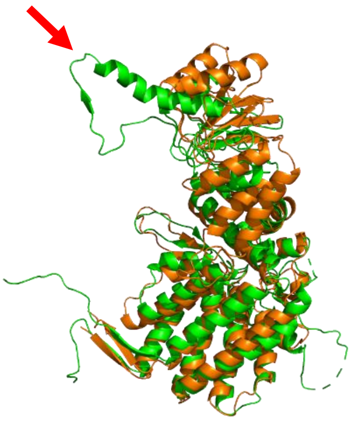

Figure S7 Comparison of MmoG (orange) based on PDB: 4V4O and eukaryotic chaperonin PDB: 4V81[9] (green) with a build-in lid (red arrow).

```
McBathOrf1          MTTEQFPPQFLREMIEQLDASIQELARKEKGLAASLGTGRVAELKEYWDHVLTPEEEWEL     60
MjNIOrf2            --MKESDFEFIDEMVSQLEGTIENLVQEERRLADKIGHARVEELKCYWQGELEPDEIEEF     58
MsiBL2Orf2          --MDEDVEQFLAAMAKSADATTHELEIEEAALRALIGGARAEELELLWTQQLDPADEEDI     58
MspOrf1             --MGPEDRDFIEEAARALDASLRELAEEEERLKEVIGEERVRELAAYLRREFEPVDIEEI     58
MmiHT12Orf1         --MNEADQEFLHDMVKQLDDTIRQLAIEESQLMGKIGAGRVEELLEYWRLELSAEEEVEF     58
MmMC09MmoE          --MKADDQEFIENMVIELDESIRRLVEEERRLKLKLGEDRVAELREFWHKQMPESEEESF     58
                         :*:       : : ..*  :*  *   :*  *. **       :   :  .:

McBathOrf1          KRTMDFRDRELVWIWSRLRRARTSRANAGEAYMRHLSPAARKNEQS------------       106
MjNIOrf2            KGGLDYWDKLIIFTWSRLNRVHETRAMAGRAIMKNNQLK---GK--------------       99
MsiBL2Orf2          KRYMDWNDKKLIWIWRRLERSRERRVAAGRAYMINSQGTAAPAISAPAKAGARPRKPL       116
MspOrf1             RRTLDFDDRRLISVWIRVERNRARRVAAGRSAMTLNAGREDIDITAFDKP----KKK-       111
MmiHT12Orf1         KKSMDYWDIQLIRILSRSKRAHNTRVEVGQTLMKMASRPILKRKPS------------       104
MmMC09MmoE          KRSMDHADRKLTWIWLRLSRLHQSRAKAGRELMKRNSID-------------------       97
                    :  :*  *  :     *  * :  *. .*.  *
```

Figure S8 Sequence alignment of MmoE (ORF1). Hypotetical proteins encoded within sMMO fragments of other methanotrophs *Methylococcus capsulatus* BATH (*Mc*Bath), *Methylomicrobium japanese* NI (MjNI), *Methylocella silvestris* BL1 (MsiBL1), *Methylosinus sporium* (*Msp*), *Methylovulum miyakonense* HT12 (*Mmi*Ht12) and MmoE from *Methylomonas methanica* MC09 (*Mm*MC09) were shown.

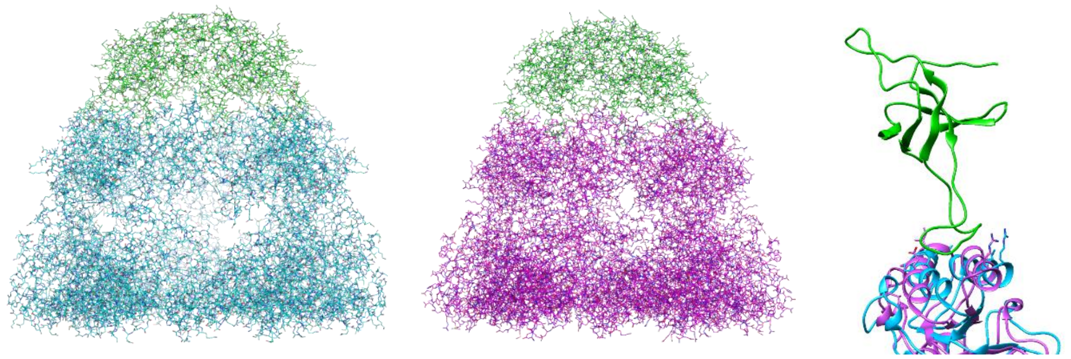

Figure S9: *In silico* docking experiments via Haddock 2.4[10] between AlphaFold2 predicted structures of GroEL (blue) and GroES (green, both left) from *M. methanica* MC09 and between MmoG (pink) and GroES (green, both middle) from *M. methanica* MC09. On the right side, an enlargement of single subunits are shown (GroES in green, GroEL in blue and MmoG in pink). The docking scores of the two structures (Haddock scoring) were -398.34 and -289.89 for MmoG/GroES and GroEL/GroES, respectively.

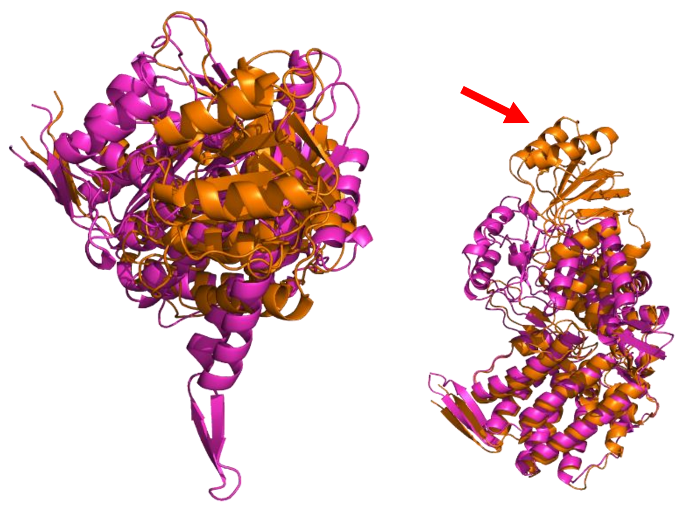

Figure S10: Structural changes between superimposed MmoG (orange) based in PDB: 4V4O with a lid (not shown, see S6) and based on PDB:6KFV) without lid (pink, see Fig S5) of single subunits. Left top view, right side view. Red arrow shows potential interface to lid, see also Fig. S7.

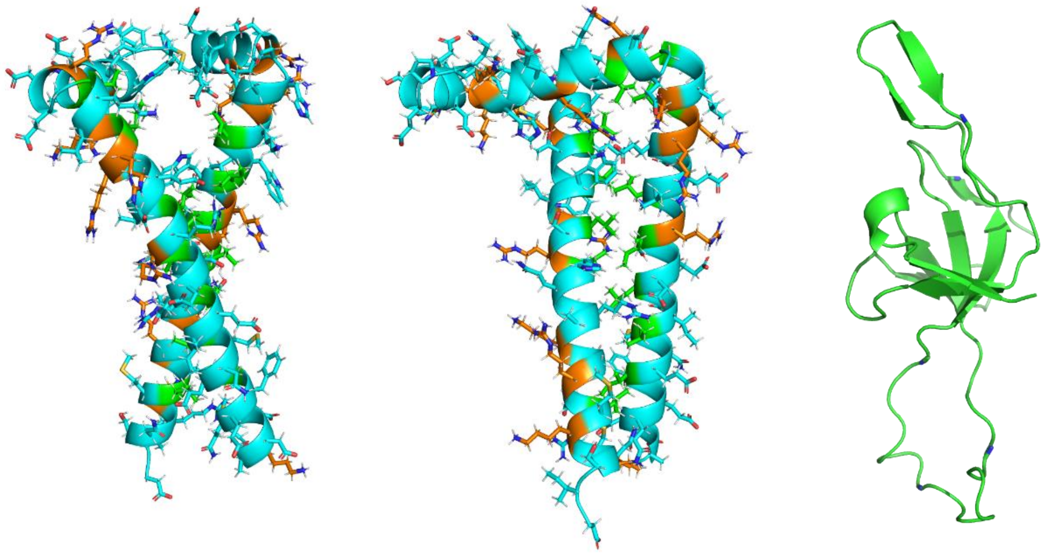

Figure S11: Predicted structure of MmoE (cyan, left and middle in different views, in green leucines, in orange lysines and arginines, which are typical for basic leucine zipper domains) based on RoseTTaFold[11] in comparison to GroES (PDB: 4V4O, right in green).

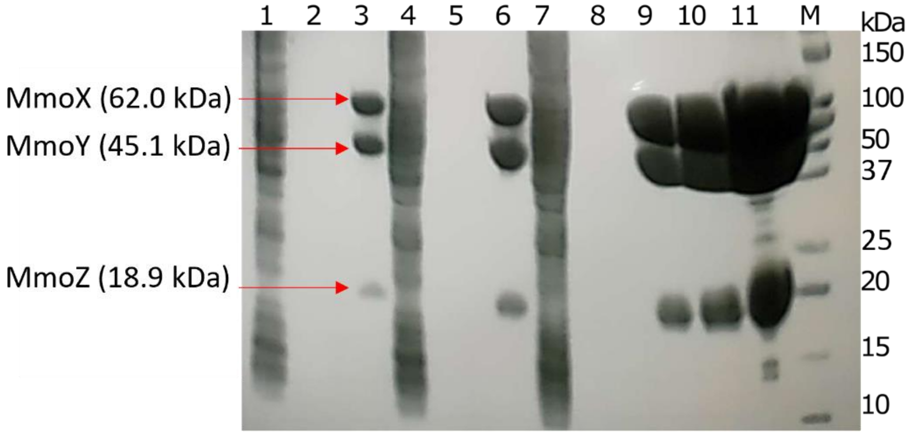

Figure S12: Evaluation of sMMO production in *E. coli* BL21 with pBB528 + pBB541 (GroESL) + pLL319 (sMMO) by SDS-PAGE gel 20 µg total protein **1, 4, 7:** 28.7 µg, 57.4 µg and 86.1 µg soluble extracts was added to each lane of a 4-15 % gradient gel, respectively; **2, 5, 8:** 15 µl wash fraction after 10 column volumes ; **3, 6, 9, 10, 11:** 11 µg, 22 µg, 44 µg, 66 µg, 132 µg purified MMOH, respectively; **M** protein marker. Cultivation in TB, sMMO production was induced with 0.1 mM IPTG, 2mM toluate overnight at 18°C. Calculated sizes of subunits are indicated.

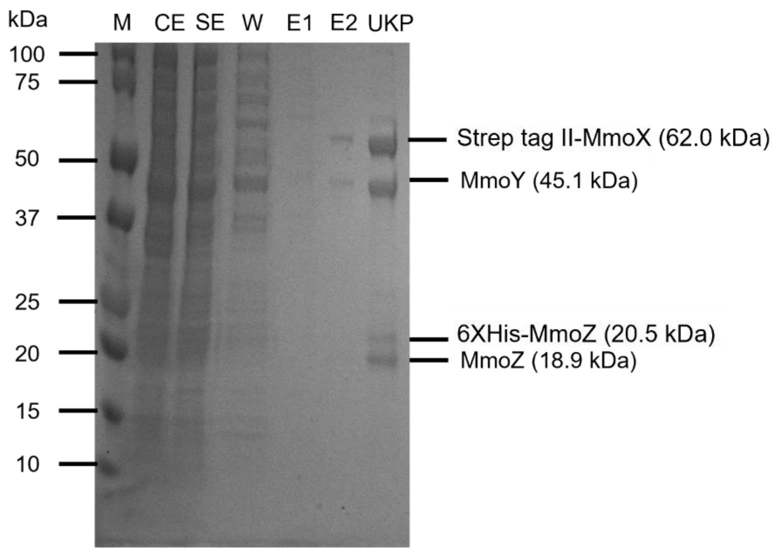

Figure S13: Purification of heterologous produced MMOH. Cell extract (CE), soluble extract (SE), one wash fraction (W), the elution fractions (E1, E2) and the buffer-exchanged and concentrated protein solution (UKP) were separated by SDS-PAGE and stained with Coomassie brilliant blue. A standard protein ladder (M) was used for size determination. For UKP and for CE/SE 2 µg and 20 µg (total protein) were applied, respectively. For W, E1 and E2 15 µl samples (max. pocket volume) were added.

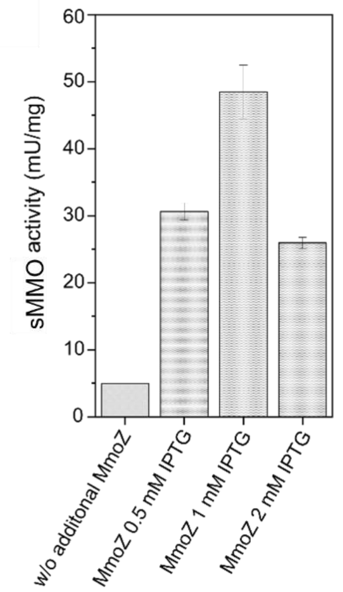

Figure S14: Influence of sMMO activity and co-synthesising MmoZ on a separate plasmid. p-nitrophenol formation from nitrobenzene was measured for sMMO without and with additional MmoZ after different inducer concentrations of IPTG to produce the proteins. Measurements were performed at optimal condition (75 mM NaCl, 37 °C and pH 8.0). The means of three technical replicates and standard deviations are shown.

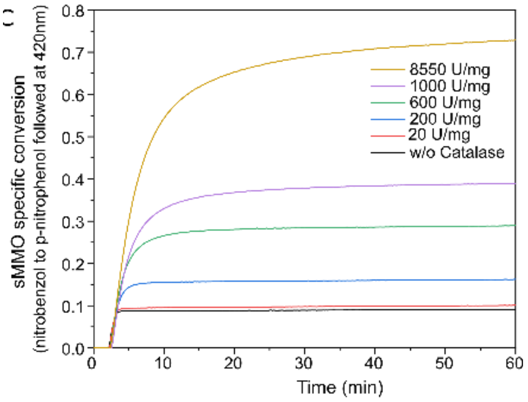

Figure S15: Adding catalase extends the period of sMMO activity. To lower the inactivation of sMMO activity due to H_2O_2 by MmoC, different catalase concentrations (0-8850 U/mg) were added to the nitrobenzene assay at optimal condition (75 mM NaCl, pH 8.0, 37 °C). MMOH was produced in *E. coli* BL21 containing the plasmids pLL319, pBB528, pBB541 and pZD04.

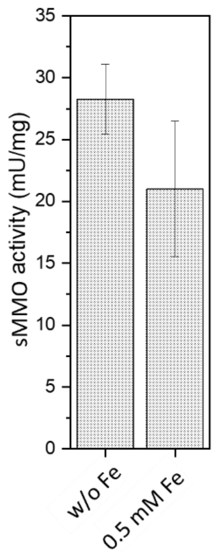

Figure S16: Iron reconstitution. To test an improvement of iron content of MMOH, a reconstitution experiment was performed. MMOH (47 µM, purified in the absence of $(NH_4)_2Fe(SO_4)_2$) was incubated for 30 min at 30 °C in iron reconstitution buffer (25 mM MOPS, 120 mM NaCl, 2 mM DTT, 5 v/v% glycerol, 0.5 mM $(NH_4)_2Fe(SO_4)_2$, pH 7.2) and then directly used for the nitrobenzene assay (25 mM MOPS, 75 mM NaCl, pH 8.0, 30 °C). Control is without addition of iron. The means of three technical replicates and standard deviations are shown.

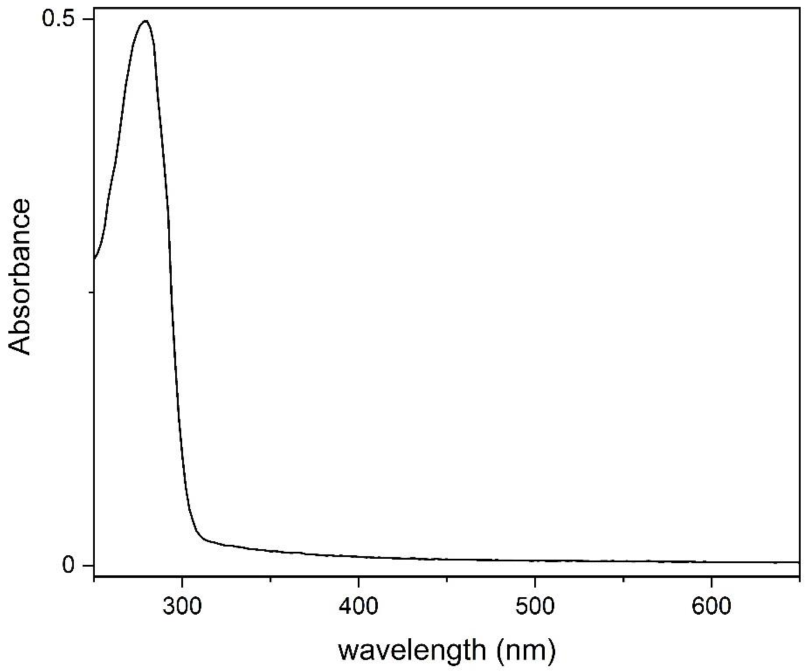

Figure S17 UV-Vis spectrum of MMOH. UV-visible absorption spectrum of purified MMOH. MMOH was stored and measured in 25 mM MOPS, pH 7.2 with 100 mM NaCl.

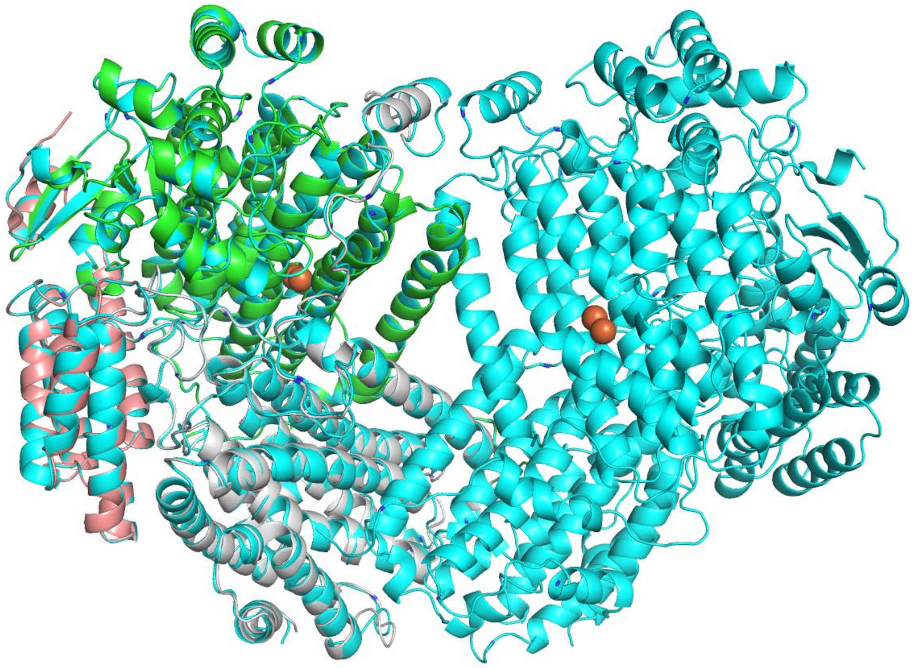

Figure S18: Homology model of MMOH including subunit MmoX (green), MmoY (grey) and MmoZ (pale red) from *M. methanica* MC09 calculated by using SWISS-MODEL[8] based on PDB: 1MTY (in cyan, homodimer), 1XMH, 7M8Q with a model quality estimation (QMEANDisCo Global) of 0.87 ± 0.05, 0.80 ± 0.07, 0.83 ± 0.07, for MmoX, MmoY, MmoZ, respectively. Brown spheres are iron atoms.

```
McBath        MALSTATKAATDALAANRAPTSVNAQEVHRWLQSFNWDFKNNRTKYATKYKMANETKEQF   60
MjNI          MAISAATKAATDALKINRAPVSVGAQEVHRWLQSFSWDFEKNRSKYPTKYHMANDTKEQF   60
MtOB3b        MAISLATKAATDALKVNRAPVGVEPQEVHKWLQSFNWDFKENRTKYPTKYHMANETKEQF   60
MsiBL1        MALSTATKAASDALGANRAPTSVSPQEVHRWLQSFNWDFAQNRTKYPTKYHMANDTKEQF   60
Msp (MmoX1)   MAISLATKAATDALKVNRAPVGVEPQEVHKWLQSFNWDFKENRTKYATKYHMANQTKEQF   60
Msp (MmoX2)   MPISLATNAATDALKVNRAPVGIEPQEVHKWLQSFNWDFKQNRTKYATKYRMANQTKEQF   60
MmiHT12       MAISAATKAATDALAVNRAPVSVGAQEVHRWMQSFTWDFEKNRTKYSTKYKMANDTKEQF   60
MmeMC09       MAISAATKAATDALAANPAPVSVGAQEVHRWMQSFTWDFDKNRTKYSTKYKMANDTKEQF   60
              * :* **:**:***  * **..:  ****:*:***.*** :**:** ***:***:*****

McBath        KLIAKEYARMEAVKDERQFGSLQDALTRLNAGVRVHPKWNETMKVVSNFLEVGEYNAIAA   120
MjNI          KLIAKEYARMESVKDERQFGSLQDALTRLDAGNRIHPKWGETMKVASNFLEVGEYNAIAA   120
MtOB3b        KVIAKEYARMEAAKDERQFGTLLDGLTRLGAGNKVHPRWGETMKVISNFLEVGEYNAIAA   120
MsiBL1        KLIAKEYARMESVKDERQFGTLLDGLTRLEAGNRVHPRWGETMKVASNFLEVGEYNAIAA   120
Msp (MmoX1)   KVIAKEYARMEAAKDERQFGTLLDGLTRLGAGNKVHPRWGETMKVISNFLEVGEYNAIAA   120
Msp (MmoX2)   KVIAKEYARMEAAKDERQFGTLLDGLTRLGAGNKIHPRWGETMKVISNFLEVGEYNAISG   120
MmiHT12       KLIAKEYARMESVKDERQFGSLQDVLTRVDAANRVHPKWNESMKVISNFLEVGEYNAIAA   120
MmeMC09       KLIAKEYARMESVKDERQFGSLQDVLTRVDAANRVHPKWNESMKVISNFLEVGEYNAIAA   120
              *:*********:.*******:* * ***: *. ::**:*.*:*** ************:.

McBath        TGMLWDSAQAAEQKNGYLAQVLDEIRHTHQCAYVNYYFAKNGQDPAGHNDARRTRTIGPL   180
MjNI          TGMLWDSATAPEQKNGYLAQVLDEIRHTNQCGYVNYYYTKHFHDPAGHNDARRTRTIGPL   180
MtOB3b        SAMLWDSATAAEQKNGYLAQVLDEIRHTHQCAFINHYYSKHYHDPAGHNDARRTRAIGPL   180
MsiBL1        SAMLWDSASAAEQKNGYLAQVLDEIRHTHQCGFVNYYFSKHYHDPAGHNDARRTRAIGPL   180
Msp (MmoX1)   SAMLWDSATAAEQKNGYLAQVLDEIRHTHQCAFINHYYSKHYHDPAGHNDARRTRAIGPL   180
Msp (MmoX2)   SAMLWDSATAAEQKNGYLAQVLDEIRHTHQCAFINHYYSKHYHDPAGHNDARRTRAIGPL   180
MmiHT12       TGMLWDSATAPEQKNGYLGQVLDEVRHTNQCGYINYYFAKQGQDAAGHNDARRTRAIGPL   180
MmeMC09       TGMLWDSATAPEQKNGYLGQVLDEIRHTNQCAYINYYFAKQGQDAAGHNDARRTRAIGPL   180
              :.****** * *******.*****:***:**.::*:*::*: :* **********:****

McBath        WKGMKRVFSDGFISGDAVECSLNLQLVGEACFTNPLIVAVTEWAAANGDEITPTVFLSIE   240
MjNI          WKGMKRVFSDGFISGDAVECSINLQLVGEACFTNPLIVAITEWAAANGDEITPTVFLSIE   240
MtOB3b        WKGMKRVFADGFISGDAVECSVNLQLVGEACFTNPLIVAVTEWASANGDEITPTVFLSVE   240
MsiBL1        WKGMKRVFADGFISGDAVECSVNLQLVGEACFTNPLIVAVTEWASANGDEITPTVFLSIE   240
Msp (MmoX1)   WKGMKRVFADGFISGDAVECSVNLQLVGEACFTNPLIVAVTEWASANGDEITPTVFLSVE   240
Msp (MmoX2)   WKGMKRVFSDGFISGDAVECSINLQLVGEACFTNPLIVAVTEWASANGDEITPTVFLSIE   240
MmiHT12       WKGMKRVFSDGFISGDAVECSINLQLVGEACFTNPLIVAVTEWASANGDEMTPTVFLSIE   240
MmeMC09       WKGMKRVFSDGFISGDAVECSINLQLVGEACFTNPLIVAVTEWASANGDEMTPTVFLSIE   240
              ********:************:*****************:****:*****:*******:*

McBath        TDELRHMANGYQTVVSIANDPASAKYLNTDLNNAFWTQQKYFTPVLGMLFEYGSKFKVEP   300
MjNI          TDELRHMANGYQTVVSIANDEAASKYLNTDLNNAFWTQQKYFTPVLGMLFEYGSKFKVEP   300
MtOB3b        TDELRHMANGYQTVVSIANDPASAKFLNTDLNNAFWTQQKYFTPVLGYLFEYGSKFKVEP   300
MsiBL1        TDELRHMANGYQTVVSIANDPAAQKYLNTDLNNAFWTQQKYFTPVLGMLFEYGSKFKVEP   300
Msp (MmoX1)   TDELRHMANGYQTVVSIANDPAAAKYLNTDLNNAFWTQQKYFTPALGYLFEYGSKFKVEP   300
Msp (MmoX2)   TDELRHMANGYQTIVSIANDPAAAKYLNTDLNNAFWTQQKYFTPALGYLFEYGSKFKVEP   300
MmiHT12       TDELRHMANGYQTVVSIANDAAASKYLNTDLNNAFWTQQKYFTPVLGMMFEYGSHFKVEP   300
MmMC09        TDELRHMANGYQTVVSIANDEAASKYLNTDLNNAFWTQQKYFTPVLGMMFEYGSHFKVEP   300
              *************:****** *: *:******************.** :*****:*****

McBath        WVKTWNRWVYEDWGGIWIGRLGKYGVESPRSLKDAKQDAYWAHHDLYLLAYALWPTGFFR   360
MjNI          WVKTWNRWVYEDWGGIWIGRLGKYGVESPRSLRDAKKDAYWAHHDLFLLAYALWPTGFFR   360
MtOB3b        WVKTWNRWVYEDWGGIWIGRLGKYGVESPASLRDAKRDAYWAHHDLALAAYAMWPLGFAR   360
MsiBL1        WVKTWNRWVYEDWGGIWIGRLAKYGVNSPPSLRDAKKDAYWAHHDLFLLAYALWPTGFFR   360
Msp (MmoX1)   WVKTWNRWVYEDWGGIWIGRLGKYGVESPRSLRDAKTDAYWAHHDLALAAYALWPLGFAR   360
Msp (MmoX2)   WVKTWNRWVYEDWGGIWIGRLGKYGVESPASLRDAKRDAYWAHHDLALAAFALWPLGFSR   360
MmiHT12       WVKTWNRWVYEDWGGIWIGRLGKYGVQSPASLRDAKKDAYWAHHDLFMIAYALWPTGFFR   360
MmMC09        WVKTWNRWVYEDWGGIWIGRLGKYGVESPRSLRDAKKDAYWAHHDLFLIAYALWPTGFFR   360
              *********************.****:** **:*** ********* : *:*:** ** *

McBath        LALPDQEEMEWFEANYPGWYDHYGKIYEEWRARGCEDPSSGFIPLMWFIENNHPIYIDRV   420
MjNI          LSLPTQEEMDWYEANYPGWYDHYGKIYEEWRARGCEDPNSGFIPLMWFIENNHQIYIDRV   420
MtOB3b        LALPDEEDQAWFEANYPGWADHYGKIFNEWKKLGYEDPKSGFIPYQWLLANGHDVYIDRV   420
MsiBL1        LSLPDEEDMEWFEANYPGWDAHYGKILREWKALGCEDPKSGFLPIQWLVQNGHQVYVDRV   420
Msp (MmoX1)   LALPDEEDQEWFEANYPGWADHYGKIYNEWKKLGYEDPKSGFIPYAWLLANGHDVYIDRV   420
Msp (MmoX2)   VSLPDEQDQEWFEANYPGWADHYGKIYNEWKRLGYEDPKSGFVPYAWLLQNGHEVYVDRV   420
MmiHT12       LTLPTAEEAEWYEANHPGWYDMYGKVYEEWKARGCEDPNSGFLPLQWFIENNHPIYIDRV   420
MmMC09        LSLPTPEEAEWYEANYPGWYDMYGKVYDEWRARGCEDPNSGFLPLQWFIENNHPIYIDRV   420
              ::**  ::  *:***:***   ***:  **:  * ***.***:*  *:: *.* :*:***
```

```
McBath       SQVPFCPSLAKGASTLRVHEYNGQMHTFSDQWGERMWLAEPERYECQNIFEQYEGRELSE   480
MjNI         SQVPFCPSLCKGASTLRVHELNGKKHSFSDDWGERMWLMEPERYECQNMFEQYAGRELSE   480
MtOB3b       SQVPFIPSLAKGTGSLRVHEFNGKKHSLTDDWGERQWLIEPERYECHNVFEQYEGRELSE   480
MsiBL1       SQVPFCPTLAKCSGSLRVHEFNGQKHSFSDDWGERMWLSEPERYECQSVFEQYSGRELSD   480
Msp (MmoX1)  SQVPFIPSLAKGSGSLRVHEFNGKKHSLTDDWGERMWLSEPERYECHNLFEQYEGRELSE   480
Msp (MmoX2)  SQVPFIPTLAKGSGSLRVHEYNGKKHSLTDDWGERQWLSEPERYEAQNLFEQYLDRELSD   480
MmiHT12      SQVPFCPSYCKGASTLRVLEFNGKKHSFSDQWGERMWLSEPERYECQNIWEQYEGRELSE   480
MmMC09       SQVPFCPSYCKGESTLRVLEYNGKKHSFSDQWGERMWLSEPERYECQNIFEQYEGRELSE   480
             ***** *: .*  .:*** * **: *:::*:**** ** ******.:.::*** .****:

McBath       VIAELHGLRSDGKTLIAQPHVR-GDKLWTLDDIKRLNCVFKNPVKAFN      527
MjNI         VIAEGHGVRSDGKTLIAQPHTDKNGKLWTLDDIKKLNCVFKDPLA---      525
MtOB3b       VIAEGHGVRSDGKTLIAQPHTR-GDNLWTLEDIKRAGCVFPDPLAKF-      526
MsiBL1       VIVEGHGVRADGKTLIGQPHVA-GSNLWTVEDLKRANCVFADPLAGF-      526
Msp (MmoX1)  VIAEGHGVRSDGKTLIAQPHVR-GDNLWTLEDIKRAGCVFPNPLAKF-      526
Msp (MmoX2)  VIAEGHGVRSDGKTLIAQPHVR-GDNLWTLEDIERAGCVFHDPLAAFG      527
MmiHT12      VIAEGHGVRSDGKTLISQPHTNKDGRLWTLDDIKKINCVFSDPLKAL-      527
MmMC09       VIAEGFGVRSDGKTLISQPHTNKDGKLWTLDDIKKINCVFSDPVKAL-      527
             **.* .*:*:******.***.  ...***::*::: .*** :*:
```

Figure S19. Sequence alignment of MmoX. Sequences from *Methylococcus capsulatus* BATH (*Mc*Bath), *Methylomicrobium japanese* NI (*Mj*NI), *Methylosinus trichosporium* OB3b (*Mt*OB3b), *Methylocella silvestris* BL1 (*Msi*BL1), *Methylosinus sporium* (*Msp*), *Methylovulum miyakonense* HT12 *(Mmi*Ht12) and *Methylomonas methanica* MC09 (*Mm*MC09) were shown. Black framed amino acids (AA) represent conserved glutamates and histidines coordinating the diiron active site. Conserved glutamates and aspartates at the surface are indicated in orange. According to the homology model (Figure S18), proposed additional negative charged amino acids on the surface of *Mme*MC09 MmoX are shown in red.

```
McBath          ---MSMLGERRRGLTDPEMAAVILKALPEAPLDGNNKMGYFVTPRWKRLTEYEALTVYAQ   57
MjNI            --MSIQVQQGKRGLTDPDMVKQILDAIPDQSLDTQRKMNYFVKPRGRRLSEYEILTCYSQ   58
MtOB3b          MSQPQSSQVTKRGLTDPERAAIIAAAVPDHALDTQRKYHYFIQPRWKPLSEYEQLSCYAQ   60
MsiBL1          ---MAIASTTKRGLTDPDKAAQILAAVPDHELDTQRRMNYFVTPRWKRLSEYEILTLYTQ   57
Msp             MSQPQSSQVTKRGLTDPERAAIIAAAVPDHALDTQRKYHYFIQPRWKRLSEYEQLSCYAQ   60
MmiHT12         --MSIEVSGGRRGLTDPALAATILAAIPDQPLETQRKMNYFMTPRGKRINEYEVLCCYTQ   58
MmeMC09         --MSIEVNGGRRGLTDPEIAAVIMAAVPEKPLETQRKMNYFMKPRGKRINEYEVLCCYSQ   58
                          :******  .  *  *:*:  *: :.:  **: ** : :.*** *  *:*

McBath          PNADWIAGGLDWGDWTQKFHGGRPSWGNETTELRTVDWFKHRDPLRRWHAPYVKDKAEEW   117
MjNI            PTPDWIPGGLDWGDWTQKFHGGRPSWGNETTELQSTDWHKHRDPAKRWHAPYVKDKAEEW   118
MtOB3b          PNPDWIAGGLDWGDWTQKFHGGRPSWGNESTELRTTDWYRHRDPARRWHHPYVKDKSEEA   120
MsiBL1          PNPDWIAGGLDWGDWTQKFHGGRPSWGNESTELRTTDWYRHRDPARRWHAPYVKDKAEEW   117
Msp             PNPDWIAGGLDWGDWTQKFHGGRPSWGNESTELRTTDWYRHRDPARRWHAPYVKDKSEEA   120
MmiHT12         PTPDWIPGGLDWGDWTQKFHGGRPSWSNESTEMRSPDWLKHRDPAFRWHALYVKDKAEEW   118
MmMC09          PTPDWIPGGLDWGDWTQKFHGGRPSWSNESTEMRSSDWLAHRDPAFRWHALYVKDKAEEW   118
                *. *** *******************.**:**::: **  ****  ***  *****:**

McBath          RYTDRFLQGYSADGQIRAMNPTWRDEFINRYWGAFLFNEYGLFNAHSQGAREALSDVTRV   177
MjNI            RYTSRFLEAYSAEGQGRSIDPVWRDEVLNDYLGAFCFNEYGLFNAHSSASRDCLGDTSRM   178
MtOB3b          RYTQRFLAAYSSEGSIRTIDPYWRDEILNKYFGALLYSEYGLFNAHSSVGRDCLSDTIRQ   180
MsiBL1          RYTTRFLEGYSAEGAVRSIDPTWRDEILDKYWGALLFSEYGLFNAHSSVSRDALSDTIRS   177
Msp             RYTQRFLAAYSSEGSIRTIDAYWRDEILNKYYGALLYNEYGLFNAHSSVGRDCLSDTIRQ   180
MmiHT12         RYTDRFLKAYSADGHVRSMDPIWRDEVLGDYLGAFGFSEYGLFNAHSSVVRDCLGDTLRM   178
MmMC09          RYTDRFLKAYSADGHVRSIDPVWRDEVLGDYLGAFGFYEYGLFNSHSSVVRDCLGDTLRM   178
                *** *** .**::*  *:::  ****.:. * **: : ******:**.  *:.*.*. *

McBath          SLAFWGFDKIDIAQMIQLERGFLAKIVPGFDESTAVPKAEWTNGEVYKSARLAVEGLWQE   237
MjNI            SIAFIGLDKVDNAQMIQLERTFLAKVFPGFPESTEIPKREWTEGKIYKGAREAVQDIWQA   238
MtOB3b          TAVFAALDKVDNAQMIQMERLFIAKLVPGFDASTDVPKKIWTTDPIYSGARATVQEIWQG   240
MsiBL1          TATFAALDKVDNAQMIQLERNFLSKIVPGFPESTDGPKKVWLTDPIYRGARETVEEAWQG   237
Msp             SATFAGLDKVDNAQMIQMERLFIAKLVPGFDASTDVPKKIWTTDPIYAGARGAVEEIWQG   240
MmiHT12         SSAMIGLDKVDNAQMIQMERTFLAKLVPGFPESTDIPKNEWTKGTIFKGSREVVQQIWQE   238
MmMC09          SSAMIGLDKVDNAQMIQAERTFLSKLVPGFPESTDIPKQEWTKGPIFKGARELVQDIWQA   238
                : .: .:**:* ***** ** *::*:.***  **  **  *  . :: .:*  *:  **

McBath          VFDWNESAFSVHAVYDALFGQFVRREFFQRLAPRFGDNLTPFFINQAQTYFQIAKQGVQD   297
MjNI            TYDWNEILWSCHMVYDPLFGQFVRREFFQRLSSYYGDTLTPFFINQMQLYYHQAKDITSD   298
MtOB3b          VQDWNEILWAGHAVMIATFGQFARREFFQRLATVYGDTLTPFFTAQSQTYFQTTRGAIDD   300
MsiBL1          IQDFNEILWAVHGVYDPLFGQFARREFFGRLAAHYGDGLTPFFLNQTQTYFQTTKAAMSD   297
Msp             IQDWNEILWAGHAVYDATFGQFARREFFQRLATVYGDTLTPFFTAQSQTYFQTTRGAIED   300
MmiHT12         TYDWNEILFSGHMIYDPLFGQFVRREFFSRLSSYYGDTLTPFFINQMQLYFSQTKGITTD   298
MmMC09          TYDWNEILFSGHMICDPIFGQFVRREFFMRLSSYYGDSLTPFFINQMQLYFSQTKGITTD   298
                  *:**  :: * :    ****.***** **:  :** *****  * * *:  ::    *

McBath          LYYNCLGDDPEFSDYNRTVMRNWTGKWLEPTIAALRDFMGLFAKLP--AGTTDKEEITAS   355
MjNI            MFFYCLGDDPEFGDYNRRMLRAWTDKWLPRTVQALHDFMGIFENIPEIAGVTSKDAVEAA   358
MtOB3b          LFVYCLANDSEFGAHNRTFLNAWTEHYLASSVAALKDFVGLYAKVEKSRADRSRRRLRGA   360
MsiBL1          LFFYSLGDDPEFGDHNRTWLRAWTNKWLQKTAEALHDFLGIYAKVDKVAGVSDPAAIKAA   357
Msp             LFVYCLANDPEFGAHNRTFLNAWTEHYLARSVTALKDFVGIYAKVEKVAGATDRAGVSEA   360
MmiHT12         MFHTCLAADGQFGAYNTRLMHVWANKWLPRTITALKGFMGIFSKIPEIKGVTDKPAIEAA   358
MmMC09          MFHTCLGADVEFGDYNNRLMRAWANKWLPRTVNALKDFMAIYATIPEIEGVSDKAAIEAA   358
                ::  .*. * :*. :*   :. *: ::*  :  **:.*:.:: .:    .  .   :  :

McBath          LYRVVDDWIEDYASRIDFKADRDQIVKAVLAGLK-                          389
MjNI            LARVLDDWQTDYADKISYKFNKAEIIKNIMKGYK-                          392
MtOB3b          AASS-AIGRSITPDKIGFRVDVDQKVDAVLAGYKN                          394
MsiBL1          VGRVVNDWVEDYAKKIDFKVDAGQLIASITRDVK-                          391
Msp             LQRVFGDWKVDYADKIGFNIDVDQKVDAVLAGFKN                          395
MmiHT12         LNRVFDDWKHDFADPIGYKADTAALIKTVLTGLK-                          392
MmMC09          LIRVFADWKRDFADPIHYKVDTAALVKTVLSGLK-                          392
                             . * :. :    :  :  . *
```

Figure S20. Sequence alignment of MmoY. Sequences from *Methylococcus capsulatus* BATH (*Mc*Bath), *Methylomicrobium japanese* NI (*Mj*NI), *Methylosinus trichosporium* OB3b (*Mt*OB3b), *Methylocella silvestris* BL1 (*Msi*BL1), *Methylosinus sporium* (*Msp*), *Methylovulum miyakonense* HT12 (*Mmi*Ht12) and *Methylomonas methanica* MC09 (*Mm*MC09) were shown. Conserved glutamates and aspartates at the surface are indicated in orange. According to the homology model (Figure S18), proposed additional negative charged amino acids on the surface of *Mme*MC09 MmoY are shown in red.

```
McBath       -MAKLGIHSNDTRDAWVNKIAQLNTLEKAAEMLKQFRMDHTTPFRNSYELDNDYLWIEAK   59
MjNI         -MANYKIHDNPVRDEWVKKIGALSSLAKGVQFLKDFREQYTTPLRKSFDLELDWGWIELK   59
MtOB3b       MAKREPIHDNSIRTEWEAKIAKLTSVDQATKFIQDFRLAYTSPFRKSYDIDVDYQYIERK   60
MsiBL1       -MPNYKIHENPVRSDWLEKIAELKSVKDATAFIQDFRKKNTSPFRTSYALDVDYLFIEAK   59
Msp          MAKREPIHENSTRTEWEGKIAKLNSVDQATKFIQDFRVAYSSPFRKSYDLDVDYQYIERK   60
MmiHT12      ---MPNIHDNPKRAEWANKIAGLKTLAQGHAFLKDFRAQHVSVFKTDFSLELDWLWIELK   57
MmMC09       ---MANIHDNPTRNAWMAKIATLDTMAKAHAFLTDFRARHMSPFKTDWSLELDGLWIELK   57
                   **.*  *  *  **. * :: ..  :: :**    : ::..: :: *  :** *

McBath       LEEKVAVLKARAFNEVDFRHKTAFGEDAKSVLDGTVAKMNAAKDKWEAEKIHIGFRQAYK   119
MjNI         IEQKLALLKHKEMNDAQILNKNADGTDAQQLANQVLASMDRCNDKWEAEKIHIQFRQQWK   119
MtOB3b       IEEKLSVLKTEKLPVADLITKATTGEDRAAVEATWIAKIKAAKSKYEADGIHIEFRQLYK   120
MsiBL1       IEERLAVLKSSTFSAADLFTKATTGETAQAVSEDWIAKIDAEKDKFAAEKILITFRQLYK   119
Msp          IEERLSVLKTEKLSVADLVTKATTGEDAAAVEAAWIAKMKAAESKYAAERIHIEFRQLYK   120
MmiHT12      IEEKVAVLKQAEFSDHQLLNVCTCGTDAQKVANDALAAMAACEDMYEAERIHINFRLACK   117
MmMC09       IEEKLALLKHKEFNDSQLLNNCSCGADAQQVANAAIAKMEACEDMYEAERIHINFRLACK   117
             :*:::::**   :   ::    : *     :    :* :   :. : *: * * **   *

McBath       PPIMPVNYFLDGERQLGTRLMELRNLNYYDTPLEELRKQRGVRVVHLQSPH   170
MjNI         PPLMPVNVFQDTDRLLGNKLMELRNANYYDMPLEELRKARGVNVVTLQ---   167
MtOB3b       PPVLPVNVFLRTDAALGTVLMEIRNTDYYGTPLEGLRKEPGVKVLHLQA--   169
MsiBL1       PPVLPVNLFFKVDTYLGSRLMELRNTDYYADSLDDLRKKRGVKVLRLGNVV   170
Msp          PPVLPVNVFLRTDAALGTILMELRNTDYYATPLEGLRKERGVKVLHLQA--   169
MmiHT12      PPVMPVNVFLDTDRQLGTKLMELRNTDYYALPLEELRKARGVRVVTLQ---   165
MmMC09       PPVMPVNVFLDTDRQLGTKLMELRNTDYYALPLEALREKRGAKVIRLQ---   165
             **::*** *   :  **. ***:** :**   *: **:  *..*: *
```

Figure S21: Sequence alignment of MmoZ. Sequences from *Methylococcus capsulatus* BATH (*Mc*Bath), *Methylomicrobium japanese* NI (*Mj*NI), *Methylosinus trichosporium* OB3b (*Mt*OB3b), *Methylocella silvestris* BL1 (*Msi*BL1), *Methylosinus sporium* (*Msp*), *Methylovulum miyakonense* HT12 *(Mmi*Ht12) and *Methylomonas methanica* MC09 (*Mm*MC09) were shown. Conserved glutamates and aspartates at the surface are indicated in orange. According to the homology model (Figure S18), proposed additional negative charged amino acids on the surface of *Mme*MC09 MmoZ are shown in red.

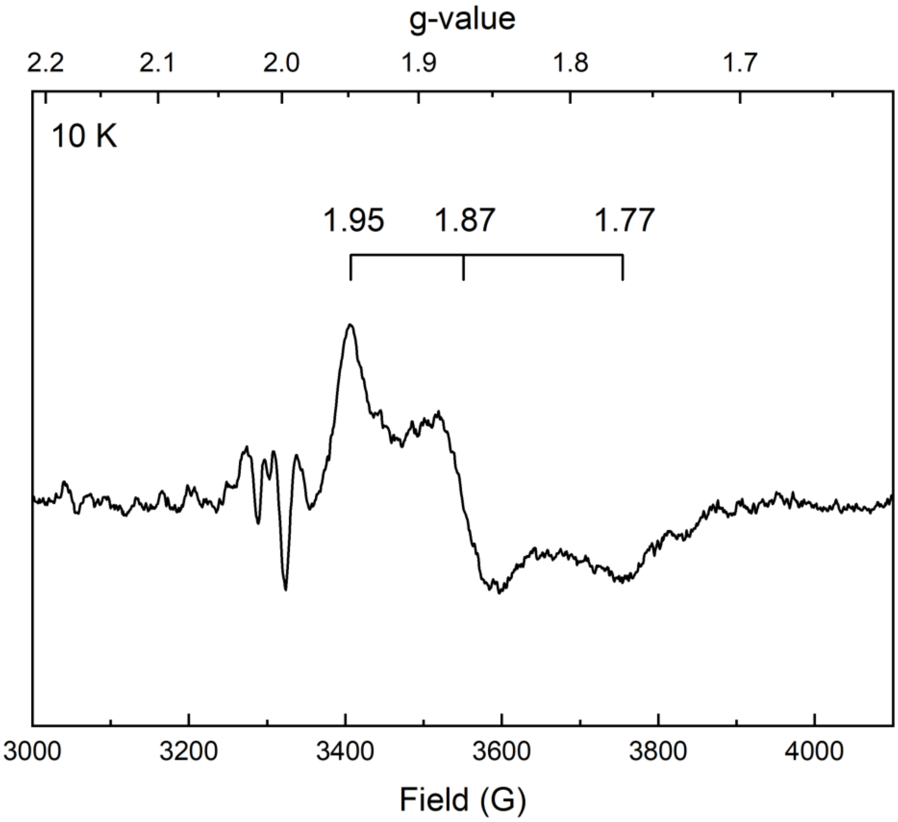

Figure S22. EPR spectrum of as isolated MMOH from *Methylomonas methanica* MC09 recorded at 10 K with a microwave power of 1 mW. The spectrum shows only a small contribution of a rhombic signal (g_x = 1.95, g_y = 1.87, g_z = 1.77), which is characteristic for the mixed-valence state of catalytic diiron center in MMOH. [12] Therefore the active site is presumably mostly in the diferric configuration. Sharp signals in the region of g = 2.0 are related to very weak artefacts of the cavity.

References:

[1] S. Balzer, V. Kucharova, J. Megerle, R. Lale, T. Brautaset, S. Valla, *Microb. Cell Factories* **2013**, *12*, 26.

[2] I. Bakke, L. Berg, T. E. V. Aune, T. Brautaset, H. Sletta, A. Tøndervik, S. Valla, *Appl. Envviron. Microbiol.* **2009**, *75*, 2002–2011.

[3] E. Lettau, D. Zill, M. Späth, C. Lorent, P. K. Singh, L. Lauterbach, *ChemBioChem* **2021**, DOI 10.1002/cbic.202100592.

[4] A. de Marco, E. Deuerling, A. Mogk, T. Tomoyasu, B. Bukau, *BMC Biotechnol.* **2007**, *7*, 1–9.

[5] L. Lauterbach, O. Lenz, *JACS* **2013**, *135*, 17897–17905.

[6] M. Neumann, S. Leimkühler, *FEBS* **2008**, *275*, 5678–5689.

[7] A. Griethe, PhD thesis „Untersuchungen zur biotechnischen Nutzung der löslichen Methan-Monooxygenase aus *Methylosinus trichosporium* Ob3b“, **2018** dx.doi.org/10.25673/13575

[8] A. Waterhouse, M. Bertoni, S. Bienert, G. Studer, G. Tauriello, R. Gumienny, F. T. Heer, T. A. P. de Beer, C. Rempfer, L. Bordoli, R. Lepore, T. Schwede, *Nucleic Acids Res.* **2018**, *46*, W296–W303.

[9] C. Dekker, S. M. Roe, E. A. McCormack, F. Beuron, L. H. Pearl, K. R. Willison, *EMBO* **2011**, *30*, 3078–3090.

[10] G. C. P. van Zundert, J. P. G. L. M. Rodrigues, M. Trellet, C. Schmitz, P. L. Kastritis, E. Karaca, A. S. J. Melquiond, M. van Dijk, S. J. de Vries, A. M. J. J. Bonvin, *J. Mol. Biol.* **2016**, *428*, 720–725.

[11] M. Baek, F. DiMaio, I. Anishchenko, J. Dauparas, S. Ovchinnikov, G. R. Lee, J. Wang, Q. Cong, L. N. Kinch, R. D. Schaeffer, C. Millán, H. Park, C. Adams, C. R. Glassman, A. DeGiovanni, J. H. Pereira, A. v. Rodrigues, A. A. van Dijk, A. C. Ebrecht, D. J. Opperman, T. Sagmeister, C. Buhlheller, T. Pavkov-Keller, M. K. Rathinaswamy, U. Dalwadi, C. K. Yip, J. E. Burke, K. C. Garcia, N. v. Grishin, P. D. Adams, R. J. Read, D. Baker, *Science* **2021**, *373*, 871–876.

[12] B. G. Fox, W. A. Froland, J. E. Dege, J. D. Lipscomb, *J. Biol. Chem.* **1989**, *264*, 10023–10033.

Figure S18: Plasmid sequence of pZD01 (*mmoB*)

TTCTTGAAGACGAAAGGGCCTCGTGATACGCCTATTTTTATAGGTTAATGTCATGATAATAATGGTTTCTTAGA
CGTCAGGTGGCACTTTTCGGGGAAATGTGCGCGGAACCCCTATTTGTTTATTTTTCTAAATACATTCAAATATGT
ATCCGCTCATGAGACAATAACCCTGATAAATGCTTCAATAATATTGAAAAAGGAAGAGTATGAGTATTCAACAT
TTCCGTGTCGCCCTTATTCCCTTTTTTGCGGCATTTTGCCTTCCTGTTTTTGCTCACCCAGAAACGCTGGTGAAAG
TAAAAGATGCTGAAGATCAGTTGGGTGCACGAGTGGGTTACATCGAACTGGATCTCAACAGCGGTAAGATCCT
TGAGAGTTTTCGCCCCGAAGAACGTTTTCCAATGATGAGCACTTTTAAAGTTCTGCTATGTGGCGCGGTATTAT
CCCGTGTTGACGCCGGGCAAGAGCAACTCGGTCGCCGCATACACTATTCTCAGAATGACTTGGTTGAGTACTC
ACCAGTCACAGAAAAGCATCTTACGGATGGCATGACAGTAAGAGAATTATGCAGTGCTGCCATAACCATGAGT
GATAACACTGCGGCCAACTTACTTCTGACAACGATCGGAGGACCGAAGGAGCTAACCGCTTTTTTGCACAACA
TGGGGGATCATGTAACTCGCCTTGATCGTTGGGAACCGGAGCTGAATGAAGCCATACCAAACGACGAGCGTG
ACACCACGATGCCTGCAGCAATGGCAACAACGTTGCGCAAACTATTAACTGGCGAACTACTTACTCTAGCTTCC
CGGCAACAATTAATAGACTGGATGGAGGCGGATAAAGTTGCAGGACCACTTCTGCGCTCGGCCCTTCCGGCTG
GCTGGTTTATTGCTGATAAATCTGGAGCCGGTGAGCGTGGGTCTCGCGGTATCATTGCAGCACTGGGGCCAGA
TGGTAAGCCCTCCCGTATCGTAGTTATCTACACGACGGGGAGTCAGGCAACTATGGATGAACGAAATAGACAG
ATCGCTGAGATAGGTGCCTCACTGATTAAGCATTGGTAACTGTCAGACCAAGTTTACTCATATATACTTTAGATT
GATTTAAAACTTCATTTTTAATTTAAAAGGATCTAGGTGAAGATCCTTTTTGATAATCTCATGACCAAAATCCCT
TAACGTGAGTTTTCGTTCCACTGAGCGTCAGACCCCGTAGAAAAGATCAAAGGATCTTCTTGAGATCCTTTTTTT
CTGCGCGTAATCTGCTGCTTGCAAACAAAAAAACCACCGCTACCAGCGGTGGTTTGTTTGCCGGATCAAGAGC
TACCAACTCTTTTTCCGAAGGTAACTGGCTTCAGCAGAGCGCAGATACCAAATACTGTCCTTCTAGTGTAGCCG
TAGTTAGGCCACCACTTCAAGAACTCTGTAGCACCGCCTACATACCTCGCTCTGCTAATCCTGTTACCAGTGGCT
GCTGCCAGTGGCGATAAGTCGTGTCTTACCGGGTTGGACTCAAGACGATAGTTACCGGATAAGGCGCAGCGG
TCGGGCTGAACGGGGGGTTCGTGCACACAGCCCAGCTTGGAGCGAACGACCTACACCGAACTGAGATACCTA
CAGCGTGAGCTATGAGAAAGCGCCACGCTTCCCGAAGGGAGAAAGGCGGACAGGTATCCGGTAAGCGGCAG
GGTCGGAACAGGAGAGCGCACGAGGGAGCTTCCAGGGGGAAACGCCTGGTATCTTTATAGTCCTGTCGGGTT
TCGCCACCTCTGACTTGAGCGTCGATTTTTGTGATGCTCGTCAGGGGGGCGGAGCCTATGGAAAAACGCCAGC
AACGCGGCCTTTTTACGGTTCCTGGCCTTTTGCTGGCCTTTTGCTCACATGTTCTTTCCTGCGTTATCCCCTGATT
CTGTGGATAACCGTATTACCGCCTTTGAGTGAGCTGATACCGCTCGCCGCAGCCGAACGACCGAGCGCAGCGA
GTCAGTGAGCGAGGAAGCGGAAGAGCGCCTGATGCGGTATTTTCTCCTTACGCATCTGTGCGGTATTTCACAC
CGCATATATGGTGCACTCTCAGTACAATCTGCTCTGATGCCGCATAGTTAAGCCAGTATACACTCCGCTATCGCT
ACGTGACTGGGTCATGGCTGCGCCCCGACACCCGCCAACACCCGCTGACGCGCCCTGACGGGCTTGTCTGCTC
CCGGCATCCGCTTACAGACAAGCTGTGACCGTCTCCGGGAGCTGCATGTGTCAGAGGTTTTCACCGTCATCACC
GAAACGCGCGAGGCAGCTGCGGTAAAGCTCATCAGCGTGGTCGTGAAGCGATTCACAGATGTCTGCCTGTTC
ATCCGCGTCCAGCTCGTTGAGTTTCTCCAGAAGCGTTAATGTCTGGCTTCTGATAAAGCGGGCCATGTTAAGGG
CGGTTTTTTCCTGTTTGGTCACTGATGCCTCCGTGTAAGGGGGATTTCTGTTCATGGGGGTAATGATACCGATG
AAACGAGAGAGGATGCTCACGATACGGGTTACTGATGATGAACATGCCCGGTTACTGGAACGTTGTGAGGGT
AAACAACTGGCGGTATGGATGCGGCGGGACCAGAGAAAAATCACTCAGGGTCAATGCCAGCGCTTCGTTAAT
ACAGATGTAGGTGTTCCACAGGGTAGCCAGCAGCATCCTGCGATGCAGATCCGGAACATAATGGTGCAGGGC
GCTGACTTCCGCGTTTCCAGACTTTACGAAACACGGAAACCGAAGACCATTCATGTTGTTGCTCAGGTCGCAGA
CGTTTTGCAGCAGCAGTCGCTTCACGTTCGCTCGCGTATCGGTGATTCATTCTGCTAACCAGTAAGGCAACCCC
GCCAGCCTAGCCGGGTCCTCAACGACAGGAGCACGATCATGCGCACCCGTGGCCAGGACCCAACGCTGCCCG
AGATGCGCCGCGTGCGGCTGCTGGAGATGGCGGACGCGATGGATATGTTCTGCCAAGGGTTGGTTTGCGCAT
TCACAGTTCTCCGCAAGAATTGATTGGCTCCAATTCTTGGAGTGGTGAATCCGTTAGCGAGGTGCCGCCGGCTT
CCATTCAGGTCGAGGTGGCCCGGCTCCATGCACCGCGACGCAACGCGGGGAGGCAGACAAGGTATAGGGCG
GCGCCTACAATCCATGCCAACCCGTTCCATGTGCTCGCCGAGGCGGCATAAATCGCCGTGACGATCAGCGGTC
CAGTGATCGAAGTTAGGCTGGTAAGAGCCGCGAGCGATCCTTGAAGCTGTCCCTGATGGTCGTCATCTACCTG
CCTGGACAGCATGGCCTGCAACGCGGGCATCCCGATGCCGCCGGAAGCGAGAAGAATCATAATGGGGAAGG

CCATCCAGCCTCGCGTCGCGAACGCCAGCAAGACGTAGCCCAGCGCGTCGGCCGCCATGCCGGCGATAATGG
CCTGCTTCTCGCCGAAACGTTTGGTGGCGGGACCAGTGACGAAGGCTTGAGCGAGGGCGTGCAAGATTCCGA
ATACCGCAAGCGACAGGCCGATCATCGTCGCGCTCCAGCGAAAGCGGTCCTCGCCGAAAATGACCCAGAGCG
CTGCCGGCACCTGTCCTACGAGTTGCATGATAAAGAAGACAGTCATAAGTGCGGCGACGATAGTCATGCCCCG
CGCCCACCGGAAGGAGCTGACTGGGTTGAAGGCTCTCAAGGGCATCGGTCGAGATCCCGGTGCCTAATGAGT
GAGCTAACTTACATTAATTGCGTTGCGCTCACTGCCCGCTTTCCAGTCGGGAAACCTGTCGTGCCAGCTGCATT
AATGAATCGGCCAACGCGCGGGGAGAGGCGGTTTGCGTATTGGGCGCCAGGGTGGTTTTTCTTTTCACCAGTG
AGACGGGCAACAGCTGATTGCCCTTCACCGCCTGGCCCTGAGAGAGTTGCAGCAAGCGGTCCACGCTGGTTTG
CCCCAGCAGGCGAAAATCCTGTTTGATGGTGGTTAACGGCGGGATATAACATGAGCTGTCTTCGGTATCGTCG
TATCCCACTACCGAGATATCCGCACCAACGCGCAGCCCGGACTCGGTAATGGCGCGCATTGCGCCCAGCGCCA
TCTGATCGTTGGCAACCAGCATCGCAGTGGGAACGATGCCCTCATTCAGCATTTGCATGGTTTGTTGAAAACCG
GACATGGCACTCCAGTCGCCTTCCCGTTCCGCTATCGGCTGAATTTGATTGCGAGTGAGATATTTATGCCAGCC
AGCCAGACGCAGACGCGCCGAGACAGAACTTAATGGGCCCGCTAACAGCGCGATTTGCTGGTGACCCAATGC
GACCAGATGCTCCACGCCCAGTCGCGTACCGTCTTCATGGGAGAAAATAATACTGTTGATGGGTGTCTGGTCA
GAGACATCAAGAAATAACGCCGGAACATTAGTGCAGGCAGCTTCCACAGCAATGGCATCCTGGTCATCCAGCG
GATAGTTAATGATCAGCCCACTGACGCGTTGCGCGAGAAGATTGTGCACCGCCGCTTTACAGGCTTCGACGCC
GCTTCGTTCTACCATCGACACCACCACGCTGGCACCCAGTTGATCGGCGCGAGATTTAATCGCCGCGACAATTT
GCGACGGCGCGTGCAGGGCCAGACTGGAGGTGGCAACGCCAATCAGCAACGACTGTTTGCCCGCCAGTTGTT
GTGCCACGCGGTTGGGAATGTAATTCAGCTCCGCCATCGCCGCTTCCACTTTTTCCCGCGTTTTCGCAGAAACG
TGGCTGGCCTGGTTCACCACGCGGGAAACGGTCTGATAAGAGACACCGGCATACTCTGCGACATCGTATAACG
TTACTGGTTTCACATTCACCACCCTGAATTGACTCTCTTCCGGGCGCTATCATGCCATACCGCGAAAGGTTTTGC
GCCATTCGATGGTGTCCGGGATCTCGACGCTCTCCCTTATGCGACTCCTGCATTAGGAAGCAGCCCAGTAGTAG
GTTGAGGCCGTTGAGCACCGCCGCCGCAAGGAATGGTGCATGCAAGGAGATGGCGCCCAACAGTCCCCCGGC
CACGGGGCCTGCCACCATACCCACGCCGAAACAAGCGCTCATGAGCCCGAAGTGGCGAGCCCGATCTTCCCCA
TCGGTGATGTCGGCGATATAGGCGCCAGCAACCGCACCTGTGGCGCCGGTGATGCCGGCCACGATGCGTCCG
GCGTAGAGGATCGAGATCTCGATCCCGCGAAATTAATACGACTCACTATAGGGGAATTGTGAGCGGATAACA
ATTCCCCTCTAGAAATAATTTTGTTTAACTTTAAGAAGGAGATATACCATGGGCCATCATCATCATCATCATCAT
CATCATCACAGCAGCGGCCATATCGAAGGTCGTCATATGAGTAAGAGTAGCAATGCCTATAATGCAGGCATTA
TGCAGAAAAATGGCAAAGCCTTTGCCGATGAATATTTTAGTGAAGATAACCAGGTGGTGCATGAAAGCCATGA
AGTTGTTCTGGTGCTGAAAAAATCAGATGAACTGAATACCGTTGTGGAAGAAATTCTGCTGGGCAGCCATAAA
GCAGATAATCCGACCCTGGTTGTGGAAGATCGTGCCGGTTTTTGGTGGCTGAAAGCAACCGGCAAAATTGAAA
TTGATTGTGTTGAAGTTAGCGAACTGCTGGGCAAACATTATAGTGTTTATGATCTGCTGGTTGATGTGAGCAGT
ACCATTGGCCGCGCATATACCCTGGGTGAAACCTTTACCATTACCAGTGAACTGATGGGCCTGGATGTGAAAC
TGAAAGATCTGGCAGCAGCCTAAGGATCCGGCTGCTAACAAAGCCCGAAAGGAAGCTGAGTTGGCTGCTGCC
ACCGCTGAGCAATAACTAGCATAACCCCTTGGGGCCTCTAAACGGGTCTTGAGGGGTTTTTTGCTGAAAGGAG
GAACTATATCCGGATATCCCGCAAGAGGCCCGGCAGTACCGGCATAACCAAGCCTATGCCTACAGCATCCAGG
GTGACGGTGCCGAGGATGACGATGAGCGCATTGTTAGATTTCATACACGGTGCCTGACTGCGTTAGCAATTTA
ACTGTGATAAACTACCGCATTAAAGCTTATCGATGATAAGCTGTCAAACATGAGAA

Figure S19: Plasmid sequence of pZD04 *(mmoZ*)

GGGGAATTGTGAGCGGATAACAATTCCCCTCTAGAAATAATTTTGTTTAACTTTAAGAAGGAGATATACCATG
GGCAGCAGCCATCACCATCATCACCACAGCCAGGATCCGATGGCCAATATTCATGATAACCCGACCCGCAATG
CCTGGATGGCCAAAATTGCCACCCTGGATACAATGGCTAAAGCCCATGCCTTCCTGACCGACTTCCGCGCCCGT
CACATGAGTCCGTTCAAAACCGATTGGAGTCTGGAACTGGATGGCCTGTGGATTGAACTGAAAATTGAAGAAA
AACTGGCACTGCTGAAACATAAAGAATTCAATGATAGCCAGCTGCTGAATAATTGCAGCTGTGGTGCCGATGC
CCAGCAGGTGGCCAATGCCGCAATTGCAAAAATGGAAGCCTGTGAAGATATGTATGAAGCAGAACGTATTCAT

ATTAACTTCCGCCTGGCCTGTAAACCGCCGGTTATGCCGGTGAATGTGTTCCTGGATACCGATCGCCAGCTGGG
TACAAAACTGATGGAACTGCGTAATACCGATTATTATGCACTGCCGCTGGAAGCCCTGCGCGAAAAACGCGGT
GCCAAAGTTATTCGCCTGCAGTAAAAGCTTGCGGCCGCATAATGCTTAAGTCGAACAGAAAGTAATCGTATTG
TACACGGCCGCATAATCGAAATTAATACGACTCACTATAGGGGAATTGTGAGCGGATAACAATTCCCCATCTTA
GTATATTAGTTAAGTATAAGAAGGAGATATACATATGGCAGATCTCAATTGGATATCGGCCGGCCACGCGATC
GCTGACGTCGGTACCCTCGAGTCTGGTAAAGAAACCGCTGCTGCGAAATTTGAACGCCAGCACATGGACTCGT
CTACTAGCGCAGCTTAATTAACCTAGGCTGCTGCCACCGCTGAGCAATAACTAGCATAACCCCTTGGGGCCTCT
AAACGGGTCTTGAGGGGTTTTTTGCTGAAAGGAGGAACTATATCCGGATTGGCGAATGGGACGCGCCCTGTA
GCGGCGCATTAAGCGCGGCGGGTGTGGTGGTTACGCGCAGCGTGACCGCTACACTTGCCAGCGCCCTAGCGC
CCGCTCCTTTCGCTTTCTTCCCTTCCTTTCTCGCCACGTTCGCCGGCTTTCCCCGTCAAGCTCTAAATCGGGGGCT
CCCTTTAGGGTTCCGATTTAGTGCTTTACGGCACCTCGACCCCAAAAAACTTGATTAGGGTGATGGTTCACGTA
GTGGGCCATCGCCCTGATAGACGGTTTTTCGCCCTTTGACGTTGGAGTCCACGTTCTTTAATAGTGGACTCTTG
TTCCAAACTGGAACAACACTCAACCCTATCTCGGTCTATTCTTTTGATTTATAAGGGATTTTGCCGATTTCGGCC
TATTGGTTAAAAAATGAGCTGATTTAACAAAAATTTAACGCGAATTTTAACAAAATATTAACGTTTACAATTTCT
GGCGGCACGATGGCATGAGATTATCAAAAAGGATCTTCACCTAGATCCTTTTAAATTAAAAATGAAGTTTTAAA
TCAATCTAAAGTATATATGAGTAAACTTGGTCTGACAGTTACCAATGCTTAATCAGTGAGGCACCTATCTCAGC
GATCTGTCTATTTCGTTCATCCATAGTTGCCTGACTCCCCGTCGTGTAGATAACTACGATACGGGAGGGCTTAC
CATCTGGCCCCAGTGCTGCAATGATACCGCGAGACCCACGCTCACCGGCTCCAGATTTATCAGCAATAAACCAG
CCAGCCGGAAGGGCCGAGCGCAGAAGTGGTCCTGCAACTTTATCCGCCTCCATCCAGTCTATTAATTGTTGCCG
GGAAGCTAGAGTAAGTAGTTCGCCAGTTAATAGTTTGCGCAACGTTGTTGCCATTGCTACAGGCATCGTGGTG
TCACGCTCGTCGTTTGGTATGGCTTCATTCAGCTCCGGTTCCCAACGATCAAGGCGAGTTACATGATCCCCCAT
GTTGTGCAAAAAAGCGGTTAGCTCCTTCGGTCCTCCGATCGTTGTCAGAAGTAAGTTGGCCGCAGTGTTATCAC
TCATGGTTATGGCAGCACTGCATAATTCTCTTACTGTCATGCCATCCGTAAGATGCTTTTCTGTGACTGGTGAGT
ACTCAACCAAGTCATTCTGAGAATAGTGTATGCGGCGACCGAGTTGCTCTTGCCCGGCGTCAATACGGGATAA
TACCGCGCCACATAGCAGAACTTTAAAAGTGCTCATCATTGGAAAACGTTCTTCGGGGCGAAAACTCTCAAGG
ATCTTACCGCTGTTGAGATCCAGTTCGATGTAACCCACTCGTGCACCCAACTGATCTTCAGCATCTTTTACTTTCA
CCAGCGTTTCTGGGTGAGCAAAAACAGGAAGGCAAAATGCCGCAAAAAAGGGAATAAGGGCGACACGGAAA
TGTTGAATACTCATACTCTTCCTTTTTCAATCATGATTGAAGCATTTATCAGGGTTATTGTCTCATGAGCGGATA
CATATTTGAATGTATTTAGAAAAATAAACAAATAGGTCATGACCAAAATCCCTTAACGTGAGTTTTCGTTCCACT
GAGCGTCAGACCCCGTAGAAAAGATCAAAGGATCTTCTTGAGATCCTTTTTTTCTGCGCGTAATCTGCTGCTTG
CAAACAAAAAAACCACCGCTACCAGCGGTGGTTTGTTTGCCGGATCAAGAGCTACCAACTCTTTTTCCGAAGGT
AACTGGCTTCAGCAGAGCGCAGATACCAAATACTGTCCTTCTAGTGTAGCCGTAGTTAGGCCACCACTTCAAGA
ACTCTGTAGCACCGCCTACATACCTCGCTCTGCTAATCCTGTTACCAGTGGCTGCTGCCAGTGGCGATAAGTCG
TGTCTTACCGGGTTGGACTCAAGACGATAGTTACCGGATAAGGCGCAGCGGTCGGGCTGAACGGGGGGTTCG
TGCACACAGCCCAGCTTGGAGCGAACGACCTACACCGAACTGAGATACCTACAGCGTGAGCTATGAGAAAGC
GCCACGCTTCCCGAAGGGAGAAAGGCGGACAGGTATCCGGTAAGCGGCAGGGTCGGAACAGGAGAGCGCAC
GAGGGAGCTTCCAGGGGGAAACGCCTGGTATCTTTATAGTCCTGTCGGGTTTCGCCACCTCTGACTTGAGCGT
CGATTTTTGTGATGCTCGTCAGGGGGGCGGAGCCTATGGAAAAACGCCAGCAACGCGGCCTTTTTACGGTTCC
TGGCCTTTTGCTGGCCTTTTGCTCACATGTTCTTTCCTGCGTTATCCCCTGATTCTGTGGATAACCGTATTACCGC
CTTTGAGTGAGCTGATACCGCTCGCCGCAGCCGAACGACCGAGCGCAGCGAGTCAGTGAGCGAGGAAGCGG
AAGAGCGCCTGATGCGGTATTTTCTCCTTACGCATCTGTGCGGTATTTCACACCGCATATATGGTGCACTCTCA
GTACAATCTGCTCTGATGCCGCATAGTTAAGCCAGTATACACTCCGCTATCGCTACGTGACTGGGTCATGGCTG
CGCCCCGACACCCGCCAACACCCGCTGACGCGCCCTGACGGGCTTGTCTGCTCCCGGCATCCGCTTACAGACA
AGCTGTGACCGTCTCCGGGAGCTGCATGTGTCAGAGGTTTTCACCGTCATCACCGAAACGCGCGAGGCAGCTG
CGGTAAAGCTCATCAGCGTGGTCGTGAAGCGATTCACAGATGTCTGCCTGTTCATCCGCGTCCAGCTCGTTGA
GTTTCTCCAGAAGCGTTAATGTCTGGCTTCTGATAAAGCGGGCCATGTTAAGGGCGGTTTTTTCCTGTTTGGTC
ACTGATGCCTCCGTGTAAGGGGGATTTCTGTTCATGGGGGTAATGATACCGATGAAACGAGAGAGGATGCTC
ACGATACGGGTTACTGATGATGAACATGCCCGGTTACTGGAACGTTGTGAGGGTAAACAACTGGCGGTATGG
ATGCGGCGGGACCAGAGAAAAATCACTCAGGGTCAATGCCAGCGCTTCGTTAATACAGATGTAGGTGTTCCAC

AGGGTAGCCAGCAGCATCCTGCGATGCAGATCCGGAACATAATGGTGCAGGGCGCTGACTTCCGCGTTTCCA
GACTTTACGAAACACGGAAACCGAAGACCATTCATGTTGTTGCTCAGGTCGCAGACGTTTTGCAGCAGCAGTC
GCTTCACGTTCGCTCGCGTATCGGTGATTCATTCTGCTAACCAGTAAGGCAACCCCGCCAGCCTAGCCGGGTCC
TCAACGACAGGAGCACGATCATGCTAGTCATGCCCCGCGCCCACCGGAAGGAGCTGACTGGGTTGAAGGCTC
TCAAGGGCATCGGTCGAGATCCCGGTGCCTAATGAGTGAGCTAACTTACATTAATTGCGTTGCGCTCACTGCCC
GCTTTCCAGTCGGGAAACCTGTCGTGCCAGCTGCATTAATGAATCGGCCAACGCGCGGGGAGAGGCGGTTTG
CGTATTGGGCGCCAGGGTGGTTTTTCTTTTCACCAGTGAGACGGGCAACAGCTGATTGCCCTTCACCGCCTGGC
CCTGAGAGAGTTGCAGCAAGCGGTCCACGCTGGTTTGCCCCAGCAGGCGAAAATCCTGTTTGATGGTGGTTAA
CGGCGGGATATAACATGAGCTGTCTTCGGTATCGTCGTATCCCACTACCGAGATGTCCGCACCAACGCGCAGC
CCGGACTCGGTAATGGCGCGCATTGCGCCCAGCGCCATCTGATCGTTGGCAACCAGCATCGCAGTGGGAACG
ATGCCCTCATTCAGCATTTGCATGGTTTGTTGAAAACCGGACATGGCACTCCAGTCGCCTTCCCGTTCCGCTATC
GGCTGAATTTGATTGCGAGTGAGATATTTATGCCAGCCAGCCAGACGCAGACGCGCCGAGACAGAACTTAAT
GGGCCCGCTAACAGCGCGATTTGCTGGTGACCCAATGCGACCAGATGCTCCACGCCCAGTCGCGTACCGTCTT
CATGGGAGAAAATAATACTGTTGATGGGTGTCTGGTCAGAGACATCAAGAAATAACGCCGGAACATTAGTGC
AGGCAGCTTCCACAGCAATGGCATCCTGGTCATCCAGCGGATAGTTAATGATCAGCCCACTGACGCGTTGCGC
GAGAAGATTGTGCACCGCCGCTTTACAGGCTTCGACGCCGCTTCGTTCTACCATCGACACCACCACGCTGGCAC
CCAGTTGATCGGCGCGAGATTTAATCGCCGCGACAATTTGCGACGGCGCGTGCAGGGCCAGACTGGAGGTGG
CAACGCCAATCAGCAACGACTGTTTGCCCGCCAGTTGTTGTGCCACGCGGTTGGGAATGTAATTCAGCTCCGCC
ATCGCCGCTTCCACTTTTTCCCGCGTTTTCGCAGAAACGTGGCTGGCCTGGTTCACCACGCGGGAAACGGTCTG
ATAAGAGACACCGGCATACTCTGCGACATCGTATAACGTTACTGGTTTCACATTCACCACCCTGAATTGACTCTC
TTCCGGGCGCTATCATGCCATACCGCGAAAGGTTTTGCGCCATTCGATGGTGTCCGGGATCTCGACGCTCTCCC
TTATGCGACTCCTGCATTAGGAAGCAGCCCAGTAGTAGGTTGAGGCCGTTGAGCACCGCCGCCGCAAGGAAT
GGTGCATGCAAGGAGATGGCGCCCAACAGTCCCCCGGCCACGGGGCCTGCCACCATACCCACGCCGAAACAA
GCGCTCATGAGCCCGAAGTGGCGAGCCCGATCTTCCCCATCGGTGATGTCGGCGATATAGGCGCCAGCAACC
GCACCTGTGGCGCCGGTGATGCCGGCCACGATGCGTCCGGCGTAGAGGATCGAGATCGATCTCGATCCCGCG
AAATTAATACGACTCACTATA

Figure S20: Plasmid sequence of pLL210

TATGGCTAGCTGGAGCCACCCGCAGTTCGAAAAAGGCGCCACTAGTTTAATTAATGCGGTACCAAGTAAAGGA
GAAGAACTTTTCACTGGAGTTGTCCCAATTCTTGTTGAATTAGATGGTGATGTTAATGGGCACAAATTTTCTGTC
AGTGGAGAGGGTGAAGGTGATGCAACATACGGAAAACTTACCCTTAAATTTATTTGCACTACTGGAAAACTAC
CTGTTCCATGGCCAACACTTGTCACTACTTTCGCGTATGGTCTTCAATGCTTTGCGAGATACCCAGATCACATGA
AGCAGCATGACTTTTTCAAGAGTGCCATGCCCGAAGGTTATGTACAGGAAAGAACTATATTTTTCAAAGATGA
CGGGAACTACAAGACACGTGCTGAAGTCAAGTTTGAAGGTGATACCCTTGTTAATAGAATCGAGTTAAAAGGT
ATTGATTTTAAAGAAGATGGAAACATTCTTGGACACAAATTGGAATACAACTATAACTCACACAATGTATACAT
CATGGCAGACAAACAAAAGAATGGAATCAAAGTTAACTTCAAAATTAGACACAACATTGAAGATGGAAGCGTT
CAACTAGCAGACCATTATCAACAAAATACTCCAATTGGCGATGGCCCTGTCCTTTTACCAGACAACCATTACCTG
TCCACACAATCTGCCCTTTCGAAAGATCCCAACGAAAAGAGAGACCACATGGTCCTTCTTGAGTTTGTAACAGC
TGCTGGGATTACACATGGCATGGATGAACTATACAAATAAGGATCCTCTAGCTAGAGTCAGCTTTATGCTTGTA
AACCGTTTTGTGAAAAAATTTTTAAAATAAAAAAGGGGACCTCTAGGGTCCCCAATTAATTAGTAATATAATCT
ATTAAAGGTCATTCAAAAGGTCATCCACCGGATCAGCTTAGTAAAGCCCTCGCTAGATTTTAATGCGGATGTTG
CGATTACTTCGCCAACTATTGCGATAACAAGAAAAAGCCAGCCTTTCATGATATATCTCCCAATTTGTGTAGGG
CTTATTATGCACGCTTAAAAATAATAAAAGCAGACTTGACCTGATAGTTTGGCTGTGAGCAATTATGTGCTTAG
TGCATCTAACGCTTGAGTTAAGCCGCGCCGCGAAGCGGCGTCGGCTTGAACGAATTGTTAGACATTATTTGCC

GACTACCAAGGATCGGGCCTTGATGTTACCCGAGAGCTTGGCACCCAGCCTGCGCGAGCAGGGGAATTGATC
CGGTGGATGACCTTTTGAATGACCTTTAATAGATTATATTACTAATTAATTGGGGACCCTAGAGGTCCCCTTTTT
TATTTTAAAAATTTTTTCACAAAACGGTTTACAAGCATAAAGCTGACCCTCTAGCAAGCTTGCGATGCAGGTGG
CTGCTGAACCCCCAGCCGGAACTGACCCCACAAGGCCCTAGCGTTTGCAATGCACCAGGTCATCATTGACCCA
GGCGTGTTCCACCAGGCCGCTGCCTCGCAACTCTTCGCAGGCTTCGCCGACCTGCTCGCGCCACTTCTTCACGC
GGGTGGAATCCGATCCGCACATGAGGCGGAAGGTTTCCAGCTTGAGCGGGTACGGCTCCCGGTGCGAGCTGA
AATAGTCGAACATCCGTCGGGCCGTCGGCGACAGCTTGCGGTACTTCTCCCATGTGAATTTCGTGTAGTGGTC
GCCAGCAAACAGCACGACGATTTCCTCGTCGATCAGGACCTGGCAACGGGACGTTTTCTTGCCACGGTCCAGG
ACGCGGAAGCGGTGCAGCAGCGACACCGATTCCAGGTGCCCAACGCGGTCGGACGTGAAGCCCATCGCCGTC
GCCTGTAGGCGCGACAGGCATTCCTCGGCCTTCGTGTAATACCGGCCATTGATCGACCAGCCCAGGTCCTGGC
AAAGCTCGTAGAACGTGAAGGTGATCGGCTCGCCGATAGGGGTGCGCTTCGCGTACTCCAACACCTGCTGCCA
CACCAGTTCGTCATCGTCGGCCCGCAGCTCGACGCCGGTGTAGGTGATCTTCACGTCCTTGTTGACGTGGAAA
ATGACCTTGTTTTGCAGCGCCTCGCGCGGGATTTTCTTGTTGCGCGTGGTGAACAGGGCAGAGCGGGCCGTGT
CGTTTGGCATCGCTCGCATCGTGTCCGGCCACGGCGCAATATCGAACAAGGAAAGCTGCATTTCCTTGATCTGC
TGCTTCGTGTGTTTCAGCAACGCGGCCTGCTTGGCCTCGCTGACCTGTTTTGCCAGGTCCTCGCCGGCGGTTTTT
CGCTTCTTGGTCGTCATAGTTCCTCGCGTGTCGATGGTCATCGACTTCGCCAAACCTGCCGCCTCCTGTTCGAGA
CGACGCGAACGCTCCACGGCGGCCGATGGCGCGGGCAGGGCAGGGGGAGCCAGTTGCACGCTGTCGCGCTC
GATCTTGGCCGTAGCTTGCTGGACCATCGAGCCGACGGACTGGAAGGTTTCGCGGGGCGCACGCATGACGGT
GCGGCTTGCGATGGTTTCGGCATCCTCGGCGGAAAACCCCGCGTCGATCAGTTCTTGCCTGTATGCCTTCCGGT
CAAACGTCCGATTCATTCACCCTCCTTGCGGGATTGCCCCGGAATTAATTCCCCGGATCGATCCGTCGATCTTGA
TCCCCTGCGCCATCAGATCCTTGGCGGCAAGAAAGCCATCCAGTTTACTTTGCAGGGCTTCCCAACCTTACCAG
AGGGCGCCCCAGCTGGCAATTCCGGTTCGCTTGCTGTCCATAAAACCGCCCAGTCTAGCTATCGCCATGTAAGC
CCACTGCAAGCTACCTGCTTTCTCTTTGCGCTTGCGTTTTCCCTTGTCCAGATAGCCCAGTAGCTGACATTCATCC
GGGGTCAGCACCGTTTCTGCGGACTGGCTTTCTACGTGGCTGCCATTTTTGGGGTGAGGTCGTTCGCGGCCGA
GGGGCGCAGCCCCTGGGGGGATGGGGTGCCGCGTTAGCGGGCCGGGAGGGTTCGAGAAGGGGGGGCACCC
CCCTTCGGCGTGCGCGGTCACGCGCCAGGGCGCAGCCCTGGTTAAAAACAAGGTTTATAAATATTGGTTTAAA
AGCAGGTTAAAAGACAGGTTAGCGGTGGCCGAAAAACGGGCGGAAACCCTTGCAAATGCTGGATTTTCTGCC
TGTGGACAGCCCCTCAAATGTCAATAGGTGCGCCCCTCATCTGTCATCACTCTGCCCCTCAAGTGTCAAGGATC
GCGCCCCTCATCTGTCAGTAGTCGCGCCCCTCAAGTGTCAATACCGCAGGGCACTTATCCCCAGGCTTGTCCAC
ATCATCTGTGGGAAACTCGCGTAAAATCAGGCGTTTTCGCCGATTTGCGAGGCTGGCCAGCTCCACGTCGCCG
GCCGAAATCGAGCCTGCCCCTCATCTGTCAACGCCGCGCCGGGTGAGTCGGCCCCTCAAGTGTCAACGTCCGC
CCCTCATCTGTCAGTGAGGGCCAAGTTTTCCGCGTGGTATCCACAACGCCGGCGGCCCTACATGGCTCTGCTGT
AGTGAGTGGGTTGCGCTCCGGCAGCGGTCCTGATCCCCCGCAGAAAAAAAGGATCTCAAGAAGATCCTTTGAT
CTTTTCTACGGGGTCTGACGCTCAGTGGAACGAAAACTCACGTTAAGGGATTTTGGTCATGAGATTATCAAAA
AGGATCTTCACCTAGATCCTTTTAAATTAAAAATGAAGTTTTAAATCAATCTAAAGTATATATGAGTAAACTTGG
TCTGACAGTTACCAATGCTTAATCAGTGAGGCACCTATCTCAGCGATCTGTCTATTTCGTTCATCCATAGTTGCC
TGACTCCCCGTCGTGTAGATAACTACGATACGGGAGGGCTTACCATCTGGCCCCAGTGCTGCAATGATACCGC
GAGACCCACGCTCACCGGCTCCAGATTTATCAGCAATAAACCAGCCAGCCGGAAGGGCCGAGCGCAGAAGTG
GTCCTGCAACTTTATCCGCCTCCATCCAGTCTATTAATTGTTGCCGGGAAGCTAGAGTAAGTAGTTCGCCAGTT
AATAGTTTGCGCTGCAGGGGGGGGGGGGGCGCTGAGGTCTGCCTCGTGAAGAAGGTGTTGCTGACTCATACCA
GGCCTGAATCGCCCCATCATCCAGCCAGAAAGTGAGGGAGCCACGGTTGATGAGAGCTTTGTTGTAGGTGGA
CCAGTTGGTGATTTTGAACTTTTGCTTTGCCACGGAACGGTCTGCGTTGTCGGGAAGATGCGTGATCTGATCCT
TCAACTCAGCAAAAGTTCGATTTATTCAACAAAGCCGCCGTCCCGTCAAGTCAGCGTAATGCTCTGCCAGTGTT
ACAACCAATTAACCAATTCTGATTAGAAAAACTCATCGAGCATCAAATGAAACTGCAATTTATTCATATCAGGA
TTATCAATACCATATTTTTGAAAAAGCCGTTTCTGTAATGAAGGAGAAAACTCACCGAGGCAGTTCCATAGGAT
GGCAAGATCCTGGTATCGGTCTGCGATTCCGACTCGTCCAACATCAATACAACCTATTAATTTCCCCTCGTCAAA
AATAAGGTTATCAAGTGAGAAATCACCATGAGTGACGACTGAATCCGGTGAGAATGGCAAAAGCTTATGCATT
TCTTTCCAGACTTGTTCAACAGGCCAGCCATTACGCTCGTCATCAAAATCACTCGCATCAACCAAACCGTTATTC
ATTCGTGATTGCGCCTGAGCGAGACGAAATACGCGATCGCTGTTAAAAGGACAATTACAAACAGGAATCGAAT

GCAACCGGCGCAGGAACACTGCCAGCGCATCAACAATATTTTCACCTGAATCAGGATATTCTTCTAATACCTGG
AATGCTGTTTTCCCGGGGATCGCAGTGGTGAGTAACCATGCATCATCAGGAGTACGGATAAAATGCTTGATGG
TCGGAAGAGGCATAAATTCCGTCAGCCAGTTTAGTCTGACCATCTCATCTGTAACATCATTGGCAACGCTACCT
TTGCCATGTTTCAGAAACAACTCTGGCGCATCGGGCTTCCCATACAATCGATAGATTGTCGCACCTGATTGCCC
GACATTATCGCGAGCCCATTTATACCCATATAAATCAGCATCCATGTTGGAATTTAATCGCGGCCTCGAGCAAG
ACGTTTCCCGTTGAATATGGCTCATAACACCCCTTGTATTACTGTTTATGTAAGCAGACAGTTTTATTGTTCATG
ATGATATATTTTTATCTTGTGCAATGTAACATCAGAGATTTTGAGACACAACGTGGCTTTCCCCCCCCCCCCTGC
AGGTCGACGGATCTTTTCCGCTGCATAACCCTGCTTCGGGGTCATTATAGCGATTTTTTCGGTATATCCATCCTT
TTTCGCACGATATACAGGATTTTGCCAAAGGGTTCGTGTAGACTTTCCTTGGTGTATCCAACGGCGTCAGCCGG
GCAGGATAGGTGAAGTAGGCCCACCCGCGAGCGGGTGTTCCTTCTTCACTGTCCCTTATTCGCACCTGGCGGT
GCTCAACGGGAATCCTGCTCTGCGAGGCTGGCCGATAAGCTCTAAGAAACCATTATTATCATGACATTAACCTA
TAAAAATAGGCGTATCACGAGGCCCTTTCGTCTTCAAGAATTAATTCACTGGCCGTCGTTTTACAACGTCGTGA
CTGGGAAAACCCTGGCGTTACCCAACTTAATCGCCTTGCAGCACATCCCCCTTTCGCCAGCAGATCCACATCCTT
GAAGGCCGCAGCGACGAGCAGAAGGAAACCCTCATTCGGGAAGTCAGCGAGGCCATCTCGCGCTCCCTGGAT
GCGCCGCTGACCAGCGTGCGAGTGATTATCACGGAGATGGCCAAGGGCCACTTCGGCATCGGCGGCGAACTG
GCCAGCAAGGTCAGACGCTGAAGTGGAGATGCCCAAGGGCACTTCGGGTCGAGGAACCCGACCTGCATTGG
GACGCGGCCACGGAGAGCGCGGGCAAACGCCGGCACTATAGCCAGTGGAGTTTGTAAAACGCTATTTCAGAG
CTTGGAGAGTGTCTAAGAAAGCCGGGCGATGCCAACCGGTCCCTTCTTCGGCTACGTTCGTAATCAAGCCACTT
CCTTTTTGCATTGACGCAGGGTGTCGGAAGGCAACTCGCCGAACGCGCTCCTATAGTTTTCAGCGAAGCGTCCC
AAATGTAAGAAGCCGTAGTCTAGGGCTATCTCAGTTATACTACGCACATTGGCACTGGGATCGTTCAAGCAGG
CGCGGATGCTTTCGAGCTTGCGGTTGCGGATGTAGTTCTTCGGCGTGGTGCCGGCATGCTTCTCGAACAAATT
GTAGAGCGAGCGTGGACTCATCATCGCCAGCTCCGCTAACCGCTCAAGGCTGATATTCCGTTTGAGATTCTCCT
CAATGAATTGAACGACTCGCTCGAAAGACGGGTTACCTTTGCTGAAAATTTCACGGCTGACATTGCTGCCCAGC
ATTTCGAGCAGCTTGGAAGCGATGATCCCCGCATAGTGCTCTTGGACCCGAGGCATCGACTTTGTATGTTCCGC
TTCGTCACAAACTAACCCGAGTAGATTGATAAAGCCATCGAGTTGCTGGAGATTGTGTCGCGCGGCGAAACGG
ATACCCTCCCTCGGCTTGTGCCAATTGTTGTCACTGCATGCCCGATCAAGGACCACTGAGGGCAATTTAACGAT
AAATTTCTCGCAATCTTCTGAATAGGTCAGGTCGGCTTGGTCATCCGGATTGAGCAGCAATAGTTCGCCCGGCG
CAAAATAGTGCTCCTGGCCATGGCCACGCCACAGGCAATGGCCTTTGAGTATTATTTGCAGATGATAACAGGT
CTCTAATCCAGGCGAGATTACCCTCACGCTACCGCCGTAGCTGATTCGACACAGGTCGAGGCATCCGAAGATT
CTGTGGTGCAGCCTGCCTGCCGGGCGCCCGCCCTTGGGCAGGCGAATAGAGTGCGTACCGACATACTGGTTA
ACATAATCGGAGACTGCATAGGGCTCGGCGTGGACGAAGATCTGACTTTTCTCGTTCAATAAGCAAAAATCCA
TAGTTCACGGTTCTCTTATTTTAATGTGAGCTCTTGGTGTGATGTAGAAAGGCGCCAAGTCGATGAAAATGCAG
GAATTAATTCGAGATCCCCCCCTGGCGGATGAGAGAAGATTTTCAGCCTGATACAGATTAAATCAGAACGCAG
AAGCGGTCTGATAAAACAGAATTTGCCTGGCGGCAGTAGCGCGGTGGTCCCACCTGACCCCATGCCGAACTCA
GAAGTGAAACGCCGTAGCGCCGATGGTAGTGTGGGGTCTCCCCATGCGAGAGTAGGGAACTGCCAGGCATCA
AATAAAACGAAAGGCTCAGTCGAAAGACTGGGCCTTTCGTTTTATCTGTTGTTTGTCGGTGAACGCTCTCCTGA
GTAGGACAAATCCGCCGGGAGCGGATTTGAACGTTGCGAAGCAACGGCCCGGAGGGTGGCGGGCAGGACGC
CCGCCATAAACTGCCAGGCATCAAATTAAGCAGAAGGCCATCCTGACGGATGGCCTTTTTGCGTAGATCCGGT
CGAGGCCGGTAGCGGAGCTATCCAACGGCGGTATACCAGGAAAACACACAGCAGGTACATCAGAACAGTACC
ATGACTGAAGAACAAATAGTTTTTTCCTGATCCATAAAGCAGAACGGCCTGCTCCATGACAAATCTGGCTCCCC
AACTAATGCCCCATGCAGCCAGCATAACCAGCATAAACGTGTCCGGTTTGATAGGGATAAGTCCAGCCTTGCA
AGAAGCGGATACAGGAGTGCAAAAAATGGCTATCTCTAGAATAGCCTACCCATTAGGCTTTATCAACATGTAC
AATAATAATGGAGTCATGAACA

Figure S21: Plasmid sequence of pLL319

TATGGCTAGCTGGAGCCACCCGCAGTTCGAAAAAGGCGCCACTATGGCTATAAGTGCAGCAACAAAAGCCGCT
ACCGACGCGTTGGCCGCCAACCCGGCGCCAGTCAGCGTGGGCGCACAGGAGGTTCACAGATGGATGCAGAGT
TTTACTTGGGATTTTGATAAAAATCGAACCAAATATTCGACCAAATACAAAATGGCCAACGATACCAAGGAGC
AATTCAAGCTGATCGCGAAAGAATATGCCCGTATGGAATCGGTAAAGGACGAGCGGCAATTCGGTAGTTTGC
AAGATGTATTGACGCGGGTAGACGCAGCCAACCGCGTGCATCCGAAATGGAACGAATCGATGAAAGTGATTT
CCAATTTCCTGGAAGTGGGTGAATACAATGCAATTGCGGCGACAGGCATGCTGTGGGATTCGGCTACCGCGCC
CGAGCAAAAAAACGGCTACCTGGGCCAGGTGTTAGATGAAATTCGTCACACCAACCAATGCGCTTACATCAAC
TACTACTTCGCCAAACAAGGCCAGGATGCCGCCGGTCATAACGATGCCCGCCGCACGCGGGCGATCGGGCCG
CTGTGGAAAGGCATGAAACGGGTGTTTTCGGACGGTTTTATTTCCGGTGACGCGGTGGAATGTTCCATTAACT
TGCAATTGGTCGGCGAAGCCTGTTTTACCAATCCGTTGATAGTAGCCGTGACCGAATGGGCGTCCGCCAACGG
TGACGAAATGACGCCGACCGTATTCCTGTCGATTGAAACCGACGAGTTACGCCACATGGCCAACGGTTATCAA
ACCGTGGTATCCATCGCCAACGACGAGGCCGCTTCAAAATATCTGAACACCGATTTGAACAACGCTTTCTGGAC
CCAGCAAAAATACTTTACACCGGTATTGGGCATGATGTTCGAGTACGGCAGCCATTTCAAGGTTGAGCCGTGG
GTTAAAACCTGGAACCGCTGGGTGTATGAAGATTGGGGCGGTATCTGGATTGGCCGACTGGGCAAATACGGT
GTCGAATCGCCCCGCAGCTTGCGCGACGCCAAAAAAGACGCCTACTGGGCACATCACGACTTGTTCTTGATTG
CTTACGCCTTGTGGCCTACCGGTTTCTTCCGCTTGAGCCTGCCCACGCCCGAAGAAGCGGAATGGTATGAAGCC
AACTATCCAGGCTGGTACGACATGTACGGCAAAGTCTATGACGAATGGCGTGCACGCGGTTGCGAGGATCCG
AACAGCGGCTTTCTGCCCTTGCAATGGTTCATCGAAAACAATCATCCGATCTATATCGACCGGGTTTCTCAAGT
CCCGTTTTGCCCAAGTTATTGCAAAGGCGAAAGTACTTTGCGGGTACTGGAGTACAACGGCAAAAAACATTCC
TTCAGCGACCAATGGGGCGAAAGAATGTGGCTTTCAGAGCCCGAGCGCTATGAGTGCCAAAACATCTTCGAAC
AGTACGAAGGTCGCGAACTGTCGGAAGTCATTGCCGAAGGCTTTGGTGTGCGTAGCGACGGCAAGACTTTGA
TCAGCCAGCCGCACACCAATAAGGACGGCAAATTATGGACGCTGGACGACATCAAAAAGATTAACTGCGTGTT
TTCCGATCCAGTGAAAGCCCTGTAATCATTTCAACCATTGTTAAGGAAACAAGCTTATGTCTATAGAAGTGAAT
GGTGGAAGACGCGGCCTGACCGACCCGGAAATTGCCGCTGTAATCATGGCCGCCGTACCCGAAAAGCCTTTG
GAAACGCAACGTAAAATGAACTACTTCATGAAGCCGCGCGGTAAACGGATTAATGAATACGAAGTATTGTGTT
GCTACAGCCAGCCGACACCGGATTGGATTCCCGGCGGTCTGGACTGGGGCGACTGGACCCAAAAATTTCATG
GTGGCCGACCGTCCTGGAGTAATGAATCCACCGAAATGCGCAGCTCGGATTGGCTGGCGCACCGCGATCCGG
CTTTCCGCTGGCACGCGCTGTATGTCAAGGATAAAGCGGAAGAATGGCGCTATACCGACCGCTTCCTGAAAGC
CTATTCCGCCGACGGCCATGTGCGTTCGATAGATCCTGTCTGGCGCGACGAAGTATTGGGAGATTACCTGGGT
GCTTTCGGCTTTTACGAGTATGGCCTGTTCAATTCGCATTCCTCCGTGGTGCGCGATTGTCTGGGCGATACGTT
GCGGATGAGCAGCGCCATGATCGGTTTGGATAAAGTCGATAACGCGCAAATGATTCAAGCGGAAAGAACCTT
TTTGTCTAAATTGGTACCGGGGTTCCCTGAATCTACCGATATTCCCAAACAGGAATGGACGAAAGGCCCCATAT
TCAAAGGCGCCAGAGAATTGGTACAGGATATTTGGCAGGCGACCTATGACTGGAACGAAATTTTGTTCTCGGG
TCATATGATCTGCGATCCCATCTTCGGTCAATTTGTGCGCAGAGAGTTCTTTATGCGTTTGTCGTCGTATTACGG
CGATAGCTTGACGCCGTTTTTTATCAACCAGATGCAGTTGTATTTCTCGCAGACCAAGGGGATTACCACGGATA
TGTTCCATACCTGCCTGGGTGCTGATGTCGAATTCGGCGACTACAACAACCGTCTGATGCGGGCCTGGGCCAA
TAAATGGCTGCCCAGAACCGTCAATGCGCTGAAAGACTTCATGGCCATTTACGCCACTATTCCGGAAATCGAA
GGTGTTAGCGACAAAGCAGCCATTGAAGCGGCGCTGATTCGGGTATTCGCCGATTGGAAACGCGATTTCGCC
GATCCGATCCACTATAAAGTGGATACCGCCGCACTCGTTAAAACCGTGTTAAGCGGACTTAAATAAGGAAATA
TCATGTCTAAAAGTTCAAATGCTTACAACGCAGGCATTATGCAAAAAAACGGCAAAGCGTTTGCCGACGAGTA
CTTTTCCGAAGACAACCAAGTCGTGCATGAAAGCCACGAAGTGGTGCTGGTGTTAAAAAAATCCGATGAACTC
AACACCGTGGTTGAAGAAATATTGCTGGGTTCTCACAAAGCGGACAACCCAACGCTGGTAGTGGAAGATCGC
GCCGGTTTCTGGTGGCTGAAAGCGACCGGTAAAATCGAGATCGACTGCGTCGAGGTCTCGGAGTTATTAGGC
AAGCATTACAGCGTTTACGACCTGCTGGTCGACGTTTCCTCCACCATCGGTCGCGCCTACACACTGGGCGAAAC
CTTTACCATCACATCTGAATTGATGGGTCTTGACGTCAAACTGAAAGACTTAGCGGCAGCATAGGGGATAACA
ATGGCAAATATTCACGATAATCCTACCCGCAATGCCTGGATGGCGAAAATTGCGACGCTGGACACTATGGCGA
AGGCACATGCTTTCTTGACCGATTTCCGGGCCAGACACATGAGCCCGTTCAAAACCGACTGGTCGCTGGAGTT
GGATGGGCTTTGGATCGAGCTGAAAATTGAAGAGAAGCTGGCGTTATTGAAACACAAGGAATTCAACGACTC
CCAATTGCTCAACAATTGCAGCTGCGGCGCCGACGCCCAGCAGGTCGCCAACGCAGCCATCGCCAAAATGGAG

GCCTGTGAAGACATGTACGAAGCAGAGCGTATTCATATCAACTTCAGATTGGCCTGCAAGCCGCCTGTCATGC
CGGTCAATGTGTTTTTGGACACGGATCGCCAATTGGGCACCAAGCTGATGGAACTGCGCAACACCGACTACTA
CGCCTTGCCGTTAGAGGCCTTGCGCGAAAAACGCGGCGCGAAAGTGATTAGGTTGCAGTAAACGCCAGGCGG
AAATGCCGGCCCCGGCTCTTTATTTATGCGGAAGAGACGGGGAAGCATGTTGCGGATGGCCATGCGGGACGC
TTGGTCATCCCGTTCAGACTATTAACCGATGGTATGCAAATTCAGCGCATTCGCGTTGGCCATGACGGACAAAT
CCGCGCATTTTTGCACCTATCGATAACACCGAACAATTTCATAGAACGAGTATCATGGATTTATTCTCAAATTTT
AAGTTGGCGGTAGCGCCTGATTTTCCGAGTGAAGCCGACAATATCACCAAGCTCTACGACAGCGAACCCTATA
CCGCATTCAGCGAAGACCTTGAGTTTATGTGGCGTTGGACAATCTATAGAGACCAAAAACTGGTGCAGGAGG
GCTGCTCCTTAACCCTGGTTGCCTCGCGGCGCGCGGTTGATCATGTACTGTTTTTTTTCAGTGCATCGAATAGA
AGCCAAGCGCGAGGAGAATGAAGTGAGCGCCCATCTAATAAAAATCGTTACCCAAGAGGGCGATTCGGTTTG
TTTCGACTGTTTCGAAGACGAAGACATTGTCAGCGCCGGTTTGCGTCAGGAAATATACCTGATGACGTCCTGTC
GCGAAGGCGGCTGCGCCACCTGTAAAGGGCTTTGCACCGATGGTGATTACGAATTAGGCAAGGTGAGTTCTC
AGGCTTTACCCACCGAAGAGGAGGAAAACGGCTATGTACTGCTGTGCCGCTGCTATCCGACCTCTGATATGGT
TGTTGAAGTACCGTACACCTACGACCGTATTTCCTTTTCGCCTGTTGGCATAAGCGTTGAGGCTGAAATCGTCG
AATTGGCCAAAGTATCCAGCAATGTCATGAAGTTGCAACTGCAAAGTCCGCCGGACGAACTGAAAATTCGCTT
CGACGCCGGCCAGTTCTTTGATCTGGAAATTCCCGGAACCACCACCACACGTTCGTATTCGCCGGCCAATATTT
CAAACGATCGCGGCGAATTGGAGTTTTTAATTCGGATTGTCGATAACGGCAAGTTTTCCGGCTGGCTGCAAAA
CCAAGCTCACGTCGGCCAAAAAATTAATGTTAAAGGACCTTCCGGTATTTTCGGACTTAAGGAAAACGGCTTTA
CCCCGCGCTATTTTGTGGCGGGCGGCACCGGGTTGGCGCCGATTTTATCCATGGTCAGACGCATGCGGGAATG
GGAAGAGCCGCAAACCAGCATCATATATTTCGGTGTCAATACCGAAGCAGAAGTGTTTTATGCGGAAGAACTG
AAACGCCTGGAATCTGAAATGCCCAATCTGGGGATACGGATTTGCGTCTGGAAAGCCAGTGACGATTGGAGC
GGCGAAAAAGGCAGTGTGGTGGATGTTTTACGCCGCGATTTGCAAGGCGGCGGCGTGACCCCGGATTTGTAT
CTGTGCGGTCCGCCCGGCATGGTGGATGCCGTATACGCAGTATGCGCCGAAGCCGGAATCGCTCAAAACAAA
ATCTTCCTGGAAAAGTTTTTACCCAGCGTTAGCTAGCGGATATGAAAGCCGACGACCAGGAGTTTATTGAAAA
TATGGTCATTGAACTGGATGAAAGTATTCGTCGCCTGGTGGAAGAAGAGCGACGCTTGAAACTCAAGCTCGGC
GAAGACCGTGTCGCCGAGTTACGCGAATTCTGGCACAAACAAATGCCGGAATCGGAAGAGGAATCTTTCAAA
CGTTCAATGGATCATGCCGATCGAAAACTAACCTGGATCTGGCTAAGGCTGAGTCGACTGCATCAATCGCGCG
CCAAAGCGGGCCGTGAGCTAATGAAACGCAATTCCATAGATTAAACGCCTGTTATTAACAACGTTTTCGAATTG
GCAATACCAACGATGCAGGCCACTCGACAAACATTTCATGACAAATTACCCATGCCTACAGGAATACCATGTCA
AAACAAGTTATTTATAATCCGGAAGCGCGCCAACGATTGCTGCAGGGGATTAACGCAGTGGCCAGAGCAGCC
GGCGTCACACTGGGTAGCGCCGGTCCGGCGGTGATGATTCAACACCGTACCGAAGGCATTATGCCTATCTTCA
CACGCGACGGTGTGACCGTTGCCAACGCCATTGTGATGGAAGATCGCATCGCCGACTTGGGTGCTCGTATGTT
GCGCGATGTTGCCGGCTCGGTATCTCGCGAGGTGGGCGATGGCACGACCACCGCGATTGTGCTGGCACAGAG
TCTGGCCGCCGATGCTTTAAAAAGCGTAGCGGCAGGCTTTCATCCGTTGCAGCTACGGAAAGGTATGGAACTG
GCTTTATCGCTTGTTGAGCAGCACTTGAAAAAAACCGCCCTGACCGGTGTTACGGAGGATTGGGTGGAAAAGA
TCGCGCTGGTTGCCACCAAAGGCGAAGCCGGCGTTGGAAAGCTGCTATCGCGGGCCTTATCCGAACTGGGCG
ACGACGGCAGCTTGACCTTCCAGCTTGGGAACGGGCGCGAGGATCAGTTAGAGGTGGTGGAAGGTATACATT
ATCAACAGGGCTTTTTATCGCCCTATTTCATCACCGATAAAACCCGTGGCGAAGCCGTTTTGGAGCAACCTTAC
ATTTTGTTGTACGACCGGGAAATCGACGATTTTATCGATTTGGTACCCATTCTGGAAGAAGTGGCCGCGGAAG
GCCGCCCGTTGTTGTTGATTGCCGAAAGTGTTTCGGAAAAAGCCTTGGCGGGCTTATTACTGAATCACGTTCGC
GGCAATTTCAAGGTGGTTGCCGTCAAGCCGCCCGGTTTCGGTGACGCACGTATCAATCGCTTAAGCGACTTGG
CATTATTGACCGGCGGTACGGCTATTTTGGAGGCGCATGCCCCGCGGCTGGAACAGGTTAAACTGACTCAGCT
GGGGCAGGCGCAACGGGCGGTTATTAACGAAGGCAGCACGACTGTTATCGGTGCGGCCGGCGCCGGTGAGC
CGATACTTGAGCGTATCGGGGCTTTACGGCGGCAGCTTGCCGCCGTAAATGCCCGCAAACCCGGCGCAGGCTC
GCCCAGCGGCAATTTACATGAGGCGGAGGAATTGGAAGAACGTATCGCCGTTCTATCCGGTAAGACCGGTGC
CTATAGTGTCGGCGGTACGACGGACGTGGAAATCAAGGAACGCCTGGTGCGGATCGAAAATGCCTATCTGTC
TGCCAAAGCAGCCGTGGCCGAAGGTGTGTTGCCGGGCGGTGGCGTAGGTCTGTTCAGATGTCGGATACTATT
GAACGAAGTCATTGCCGAAAATGCCGAGCAACAGCAGGGCGTCGCCATCATCAAAAATGCGCTGGGCGCGCC
GATGCGGCAATTGTTGAGCAATGGCGGCTTGAATAGCGAAGAAGTCATGATGCGTTTGTGTGGCCAGACAGA

TCCGCAATTTGCCTTTGATATGCGGCATCAGCGTTATGGGCATTTTCTGGATATTGGCATTATAGACTCGGTAA
AAGTGGTGCGCTTGGCATTACGCAAAGCGGTGAGTGTGGTGGGCACTTTAATCAGCAGCGATACGGTGATCA
TGAATGTGCCGGACTTATCCATTATGGACGGTTACTCTGCTGAATGGGCCGCCGCTACCCGCGAAGACCCTCG
CAGTTGACGGCTCATGGTCATGTCTGATGCATATACCCCAACGGCATTGGCGATAGTGGCTGGCGGCGGAAAA
CCTCGCTGAGCGTCTCAGCAGACTTGAGTTGTTTGTGCCGGGGCAAATTTCGGTACTTGTCTCCGTCATAAGCA
TATAAACAGCCATTTAAGAAAACGAACGGCTTAACCGATTGTATTAGCTGTATCCTTCCTGCCCCGGTTGCTCTC
GCTCCAGAGTTCCTGATAATAGTATGGGGCAAGGCAATGTGACGTTATGTCACATTGCCTATTTTTTTGATTTTG
TTATTTTTGCATAGTCTAAACAGATATAAATAACTGTTTTTAAATAAGTTTCGCGCCGAAACTGTTTTGGCAAGT
CAAAATCGAGAGATTTAATTAATGCGGTACCAAGTAAAGGAGAAGAACTTTTCACTGGAGTTGTCCCAATTCTT
GTTGAATTAGATGGTGATGTTAATGGGCACAAATTTTCTGTCAGTGGAGAGGGTGAAGGTGATGCAACATAC
GGAAAACTTACCCTTAAATTTATTTGCACTACTGGAAAACTACCTGTTCCATGGCCAACACTTGTCACTACTTTC
GCGTATGGTCTTCAATGCTTTGCGAGATACCCAGATCACATGAAGCAGCATGACTTTTTCAAGAGTGCCATGCC
CGAAGGTTATGTACAGGAAAGAACTATATTTTTCAAAGATGACGGGAACTACAAGACACGTGCTGAAGTCAAG
TTTGAAGGTGATACCCTTGTTAATAGAATCGAGTTAAAAGGTATTGATTTTAAAGAAGATGGAAACATTCTTGG
ACACAAATTGGAATACAACTATAACTCACACAATGTATACATCATGGCAGACAAACAAAAGAATGGAATCAAA
GTTAACTTCAAAATTAGACACAACATTGAAGATGGAAGCGTTCAACTAGCAGACCATTATCAACAAAATACTCC
AATTGGCGATGGCCCTGTCCTTTTACCAGACAACCATTACCTGTCCACACAATCTGCCCTTTCGAAAGATCCCAA
CGAAAAGAGAGACCACATGGTCCTTCTTGAGTTTGTAACAGCTGCTGGGATTACACATGGCATGGATGAACTA
TACAAATAAGGATCCTCTAGCTAGAGTCAGCTTTATGCTTGTAAACCGTTTTGTGAAAAAATTTTTAAAATAAAA
AAGGGGACCTCTAGGGTCCCCAATTAATTAGTAATATAATCTATTAAAGGTCATTCAAAAGGTCATCCACCGGA
TCAGCTTAGTAAAGCCCTCGCTAGATTTTAATGCGGATGTTGCGATTACTTCGCCAACTATTGCGATAACAAGA
AAAAGCCAGCCTTTCATGATATATCTCCCAATTTGTGTAGGGCTTATTATGCACGCTTAAAAATAATAAAAGCA
GACTTGACCTGATAGTTTGGCTGTGAGCAATTATGTGCTTAGTGCATCTAACGCTTGAGTTAAGCCGCGCCGCG
AAGCGGCGTCGGCTTGAACGAATTGTTAGACATTATTTGCCGACTACCAAGGATCGGGCCTTGATGTTACCCG
AGAGCTTGGCACCCAGCCTGCGCGAGCAGGGGAATTGATCCGGTGGATGACCTTTTGAATGACCTTTAATAGA
TTATATTACTAATTAATTGGGGACCCTAGAGGTCCCCTTTTTTATTTTAAAAATTTTTTCACAAAACGGTTTACAA
GCATAAAGCTGACCCTCTAGCAAGCTTGCGATGCAGGTGGCTGCTGAACCCCCAGCCGGAACTGACCCCACAA
GGCCCTAGCGTTTGCAATGCACCAGGTCATCATTGACCCAGGCGTGTTCCACCAGGCCGCTGCCTCGCAACTCT
TCGCAGGCTTCGCCGACCTGCTCGCGCCACTTCTTCACGCGGGTGGAATCCGATCCGCACATGAGGCGGAAGG
TTTCCAGCTTGAGCGGGTACGGCTCCCGGTGCGAGCTGAAATAGTCGAACATCCGTCGGGCCGTCGGCGACA
GCTTGCGGTACTTCTCCCATGTGAATTTCGTGTAGTGGTCGCCAGCAAACAGCACGACGATTTCCTCGTCGATC
AGGACCTGGCAACGGGACGTTTTCTTGCCACGGTCCAGGACGCGGAAGCGGTGCAGCAGCGACACCGATTCC
AGGTGCCCAACGCGGTCGGACGTGAAGCCCATCGCCGTCGCCTGTAGGCGCGACAGGCATTCCTCGGCCTTCG
TGTAATACCGGCCATTGATCGACCAGCCCAGGTCCTGGCAAAGCTCGTAGAACGTGAAGGTGATCGGCTCGCC
GATAGGGGTGCGCTTCGCGTACTCCAACACCTGCTGCCACACCAGTTCGTCATCGTCGGCCCGCAGCTCGACG
CCGGTGTAGGTGATCTTCACGTCCTTGTTGACGTGGAAAATGACCTTGTTTTGCAGCGCCTCGCGCGGGATTTT
CTTGTTGCGCGTGGTGAACAGGGCAGAGCGGGCCGTGTCGTTTGGCATCGCTCGCATCGTGTCCGGCCACGG
CGCAATATCGAACAAGGAAAGCTGCATTTCCTTGATCTGCTGCTTCGTGTGTTTCAGCAACGCGGCCTGCTTGG
CCTCGCTGACCTGTTTTGCCAGGTCCTCGCCGGCGGTTTTTCGCTTCTTGGTCGTCATAGTTCCTCGCGTGTCGA
TGGTCATCGACTTCGCCAAACCTGCCGCCTCCTGTTCGAGACGACGCGAACGCTCCACGGCGGCCGATGGCGC
GGGCAGGGCAGGGGGAGCCAGTTGCACGCTGTCGCGCTCGATCTTGGCCGTAGCTTGCTGGACCATCGAGCC
GACGGACTGGAAGGTTTCGCGGGGCGCACGCATGACGGTGCGGCTTGCGATGGTTTCGGCATCCTCGGCGGA
AAACCCCGCGTCGATCAGTTCTTGCCTGTATGCCTTCCGGTCAAACGTCCGATTCATTCACCCTCCTTGCGGGAT
TGCCCCGGAATTAATTCCCCGGATCGATCCGTCGATCTTGATCCCCTGCGCCATCAGATCCTTGGCGGCAAGAA
AGCCATCCAGTTTACTTTGCAGGGCTTCCCAACCTTACCAGAGGGCGCCCCAGCTGGCAATTCCGGTTCGCTTG
CTGTCCATAAAACCGCCCAGTCTAGCTATCGCCATGTAAGCCCACTGCAAGCTACCTGCTTTCTCTTTGCGCTTG
CGTTTTCCCTTGTCCAGATAGCCCAGTAGCTGACATTCATCCGGGGTCAGCACCGTTTCTGCGGACTGGCTTTCT
ACGTGGCTGCCATTTTTGGGGTGAGGTCGTTCGCGGCCGAGGGGCGCAGCCCCTGGGGGGATGGGGTGCCG
CGTTAGCGGGCCGGGAGGGTTCGAGAAGGGGGGGCACCCCCCTTCGGCGTGCGCGGTCACGCGCCAGGGCG

CAGCCCTGGTTAAAAACAAGGTTTATAAATATTGGTTTAAAAGCAGGTTAAAAGACAGGTTAGCGGTGGCCGA
AAAACGGGCGGAAACCCTTGCAAATGCTGGATTTTCTGCCTGTGGACAGCCCCTCAAATGTCAATAGGTGCGC
CCCTCATCTGTCATCACTCTGCCCCTCAAGTGTCAAGGATCGCGCCCCTCATCTGTCAGTAGTCGCGCCCCTCAA
GTGTCAATACCGCAGGGCACTTATCCCCAGGCTTGTCCACATCATCTGTGGGAAACTCGCGTAAAATCAGGCG
TTTTCGCCGATTTGCGAGGCTGGCCAGCTCCACGTCGCCGGCCGAAATCGAGCCTGCCCCTCATCTGTCAACGC
CGCGCCGGGTGAGTCGGCCCCTCAAGTGTCAACGTCCGCCCCTCATCTGTCAGTGAGGGCCAAGTTTTCCGCG
TGGTATCCACAACGCCGGCGGCCCTACATGGCTCTGCTGTAGTGAGTGGGTTGCGCTCCGGCAGCGGTCCTGA
TCCCCCGCAGAAAAAAAGGATCTCAAGAAGATCCTTTGATCTTTTCTACGGGGTCTGACGCTCAGTGGAACGA
AAACTCACGTTAAGGGATTTTGGTCATGAGATTATCAAAAAGGATCTTCACCTAGATCCTTTTAAATTAAAAAT
GAAGTTTTAAATCAATCTAAAGTATATATGAGTAAACTTGGTCTGACAGTTACCAATGCTTAATCAGTGAGGCA
CCTATCTCAGCGATCTGTCTATTTCGTTCATCCATAGTTGCCTGACTCCCCGTCGTGTAGATAACTACGATACGG
GAGGGCTTACCATCTGGCCCCAGTGCTGCAATGATACCGCGAGACCCACGCTCACCGGCTCCAGATTTATCAG
CAATAAACCAGCCAGCCGGAAGGGCCGAGCGCAGAAGTGGTCCTGCAACTTTATCCGCCTCCATCCAGTCTAT
TAATTGTTGCCGGGAAGCTAGAGTAAGTAGTTCGCCAGTTAATAGTTTGCGCTGCAGGGGGGGGGGGGGCGCT
GAGGTCTGCCTCGTGAAGAAGGTGTTGCTGACTCATACCAGGCCTGAATCGCCCCATCATCCAGCCAGAAAGT
GAGGGAGCCACGGTTGATGAGAGCTTTGTTGTAGGTGGACCAGTTGGTGATTTTGAACTTTTGCTTTGCCACG
GAACGGTCTGCGTTGTCGGGAAGATGCGTGATCTGATCCTTCAACTCAGCAAAAGTTCGATTTATTCAACAAA
GCCGCCGTCCCGTCAAGTCAGCGTAATGCTCTGCCAGTGTTACAACCAATTAACCAATTCTGATTAGAAAAACT
CATCGAGCATCAAATGAAACTGCAATTTATTCATATCAGGATTATCAATACCATATTTTTGAAAAAGCCGTTTCT
GTAATGAAGGAGAAAACTCACCGAGGCAGTTCCATAGGATGGCAAGATCCTGGTATCGGTCTGCGATTCCGA
CTCGTCCAACATCAATACAACCTATTAATTTCCCCTCGTCAAAAATAAGGTTATCAAGTGAGAAATCACCATGA
GTGACGACTGAATCCGGTGAGAATGGCAAAAGCTTATGCATTTCTTTCCAGACTTGTTCAACAGGCCAGCCATT
ACGCTCGTCATCAAAATCACTCGCATCAACCAAACCGTTATTCATTCGTGATTGCGCCTGAGCGAGACGAAATA
CGCGATCGCTGTTAAAAGGACAATTACAAACAGGAATCGAATGCAACCGGCGCAGGAACACTGCCAGCGCAT
CAACAATATTTTCACCTGAATCAGGATATTCTTCTAATACCTGGAATGCTGTTTTCCCGGGGATCGCAGTGGTG
AGTAACCATGCATCATCAGGAGTACGGATAAAATGCTTGATGGTCGGAAGAGGCATAAATTCCGTCAGCCAGT
TTAGTCTGACCATCTCATCTGTAACATCATTGGCAACGCTACCTTTGCCATGTTTCAGAAACAACTCTGGCGCAT
CGGGCTTCCCATACAATCGATAGATTGTCGCACCTGATTGCCCGACATTATCGCGAGCCCATTTATACCCATATA
AATCAGCATCCATGTTGGAATTTAATCGCGGCCTCGAGCAAGACGTTTCCCGTTGAATATGGCTCATAACACCC
CTTGTATTACTGTTTATGTAAGCAGACAGTTTTATTGTTCATGATGATATATTTTTATCTTGTGCAATGTAACATC
AGAGATTTTGAGACACAACGTGGCTTTCCCCCCCCCCCCTGCAGGTCGACGGATCTTTTCCGCTGCATAACCCT
GCTTCGGGGTCATTATAGCGATTTTTTCGGTATATCCATCCTTTTTCGCACGATATACAGGATTTTGCCAAAGGG
TTCGTGTAGACTTTCCTTGGTGTATCCAACGGCGTCAGCCGGGCAGGATAGGTGAAGTAGGCCCACCCGCGAG
CGGGTGTTCCTTCTTCACTGTCCCTTATTCGCACCTGGCGGTGCTCAACGGGAATCCTGCTCTGCGAGGCTGGC
CGATAAGCTCTAAGAAACCATTATTATCATGACATTAACCTATAAAAATAGGCGTATCACGAGGCCCTTTCGTC
TTCAAGAATTAATTCACTGGCCGTCGTTTTACAACGTCGTGACTGGGAAAACCCTGGCGTTACCCAACTTAATC
GCCTTGCAGCACATCCCCCTTTCGCCAGCAGATCCACATCCTTGAAGGCCGCAGCGACGAGCAGAAGGAAACC
CTCATTCGGGAAGTCAGCGAGGCCATCTCGCGCTCCCTGGATGCGCCGCTGACCAGCGTGCGAGTGATTATCA
CGGAGATGGCCAAGGGCCACTTCGGCATCGGCGGCGAACTGGCCAGCAAGGTCAGACGCTGAAGTGGAGAT
GCCCAAGGGCACTTCGGGTCGAGGAACCCGACCTGCATTGGGACGCGGCCACGGAGAGCGCGGGCAAACGC
CGGCACTATAGCCAGTGGAGTTTGTAAAACGCTATTTCAGAGCTTGGAGAGTGTCTAAGAAAGCCGGGCGAT
GCCAACCGGTCCCTTCTTCGGCTACGTTCGTAATCAAGCCACTTCCTTTTTGCATTGACGCAGGGTGTCGGAAG
GCAACTCGCCGAACGCGCTCCTATAGTTTTCAGCGAAGCGTCCCAAATGTAAGAAGCCGTAGTCTAGGGCTAT
CTCAGTTATACTACGCACATTGGCACTGGGATCGTTCAAGCAGGCGCGGATGCTTTCGAGCTTGCGGTTGCGG
ATGTAGTTCTTCGGCGTGGTGCCGGCATGCTTCTCGAACAAATTGTAGAGCGAGCGTGGACTCATCATCGCCA
GCTCCGCTAACCGCTCAAGGCTGATATTCCGTTTGAGATTCTCCTCAATGAATTGAACGACTCGCTCGAAAGAC
GGGTTACCTTTGCTGAAAATTTCACGGCTGACATTGCTGCCCAGCATTTCGAGCAGCTTGGAAGCGATGATCCC
CGCATAGTGCTCTTGGACCCGAGGCATCGACTTTGTATGTTCCGCTTCGTCACAAACTAACCCGAGTAGATTGA
TAAAGCCATCGAGTTGCTGGAGATTGTGTCGCGCGGCGAAACGGATACCCTCCCTCGGCTTGTGCCAATTGTT

GTCACTGCATGCCCGATCAAGGACCACTGAGGGCAATTTAACGATAAATTTCTCGCAATCTTCTGAATAGGTCA
GGTCGGCTTGGTCATCCGGATTGAGCAGCAATAGTTCGCCCGGCGCAAAATAGTGCTCCTGGCCATGGCCACG
CCACAGGCAATGGCCTTTGAGTATTATTTGCAGATGATAACAGGTCTCTAATCCAGGCGAGATTACCCTCACGC
TACCGCCGTAGCTGATTCGACACAGGTCGAGGCATCCGAAGATTCTGTGGTGCAGCCTGCCTGCCGGGCGCCC
GCCCTTGGGCAGGCGAATAGAGTGCGTACCGACATACTGGTTAACATAATCGGAGACTGCATAGGGCTCGGC
GTGGACGAAGATCTGACTTTTCTCGTTCAATAAGCAAAAATCCATAGTTCACGGTTCTCTTATTTTAATGTGAGC
TCTTGGTGTGATGTAGAAAGGCGCCAAGTCGATGAAAATGCAGGAATTAATTCGAGATCCCCCCCTGGCGGAT
GAGAGAAGATTTTCAGCCTGATACAGATTAAATCAGAACGCAGAAGCGGTCTGATAAAACAGAATTTGCCTGG
CGGCAGTAGCGCGGTGGTCCCACCTGACCCCATGCCGAACTCAGAAGTGAAACGCCGTAGCGCCGATGGTAG
TGTGGGGTCTCCCCATGCGAGAGTAGGGAACTGCCAGGCATCAAATAAAACGAAAGGCTCAGTCGAAAGACT
GGGCCTTTCGTTTTATCTGTTGTTTGTCGGTGAACGCTCTCCTGAGTAGGACAAATCCGCCGGGAGCGGATTTG
AACGTTGCGAAGCAACGGCCCGGAGGGTGGCGGGCAGGACGCCCGCCATAAACTGCCAGGCATCAAATTAA
GCAGAAGGCCATCCTGACGGATGGCCTTTTTGCGTAGATCCGGTCGAGGCCGGTAGCGGAGCTATCCAACGG
CGGTATACCAGGAAAACACACAGCAGGTACATCAGAACAGTACCATGACTGAAGAACAAATAGTTTTTTCCTG
ATCCATAAAGCAGAACGGCCTGCTCCATGACAAATCTGGCTCCCCAACTAATGCCCCATGCAGCCAGCATAACC
AGCATAAACGTGTCCGGTTTGATAGGGATAAGTCCAGCCTTGCAAGAAGCGGATACAGGAGTGCAAAAAATG
GCTATCTCTAGAATAGCCTACCCATTAGGCTTTATCAACATGTACAATAATAATGGAGTCATGAACA

Figure S22: Plasmid sequence of pZD02

TTCTTGAAGACGAAAGGGCCTCGTGATACGCCTATTTTTATAGGTTAATGTCATGATAATAATGGTTTCTTAGA
CGTCAGGTGGCACTTTTCGGGGAAATGTGCGCGGAACCCCTATTTGTTTATTTTTCTAAATACATTCAAATATGT
ATCCGCTCATGAGACAATAACCCTGATAAATGCTTCAATAATATTGAAAAAGGAAGAGTATGAGTATTCAACAT
TTCCGTGTCGCCCTTATTCCCTTTTTTGCGGCATTTTGCCTTCCTGTTTTTGCTCACCCAGAAACGCTGGTGAAAG
TAAAAGATGCTGAAGATCAGTTGGGTGCACGAGTGGGTTACATCGAACTGGATCTCAACAGCGGTAAGATCCT
TGAGAGTTTTCGCCCCGAAGAACGTTTTCCAATGATGAGCACTTTTAAAGTTCTGCTATGTGGCGCGGTATTAT
CCCGTGTTGACGCCGGGCAAGAGCAACTCGGTCGCCGCATACACTATTCTCAGAATGACTTGGTTGAGTACTC
ACCAGTCACAGAAAAGCATCTTACGGATGGCATGACAGTAAGAGAATTATGCAGTGCTGCCATAACCATGAGT
GATAACACTGCGGCCAACTTACTTCTGACAACGATCGGAGGACCGAAGGAGCTAACCGCTTTTTTGCACAACA
TGGGGGATCATGTAACTCGCCTTGATCGTTGGGAACCGGAGCTGAATGAAGCCATACCAAACGACGAGCGTG
ACACCACGATGCCTGCAGCAATGGCAACAACGTTGCGCAAACTATTAACTGGCGAACTACTTACTCTAGCTTCC
CGGCAACAATTAATAGACTGGATGGAGGCGGATAAAGTTGCAGGACCACTTCTGCGCTCGGCCCTTCCGGCTG
GCTGGTTTATTGCTGATAAATCTGGAGCCGGTGAGCGTGGGTCTCGCGGTATCATTGCAGCACTGGGGCCAGA
TGGTAAGCCCTCCCGTATCGTAGTTATCTACACGACGGGGAGTCAGGCAACTATGGATGAACGAAATAGACAG
ATCGCTGAGATAGGTGCCTCACTGATTAAGCATTGGTAACTGTCAGACCAAGTTTACTCATATATACTTTAGATT
GATTTAAAACTTCATTTTTAATTTAAAAGGATCTAGGTGAAGATCCTTTTTGATAATCTCATGACCAAAATCCCT
TAACGTGAGTTTTCGTTCCACTGAGCGTCAGACCCCGTAGAAAAGATCAAAGGATCTTCTTGAGATCCTTTTTTT
CTGCGCGTAATCTGCTGCTTGCAAACAAAAAAACCACCGCTACCAGCGGTGGTTTGTTTGCCGGATCAAGAGC
TACCAACTCTTTTTCCGAAGGTAACTGGCTTCAGCAGAGCGCAGATACCAAATACTGTCCTTCTAGTGTAGCCG
TAGTTAGGCCACCACTTCAAGAACTCTGTAGCACCGCCTACATACCTCGCTCTGCTAATCCTGTTACCAGTGGCT
GCTGCCAGTGGCGATAAGTCGTGTCTTACCGGGTTGGACTCAAGACGATAGTTACCGGATAAGGCGCAGCGG
TCGGGCTGAACGGGGGGTTCGTGCACACAGCCCAGCTTGGAGCGAACGACCTACACCGAACTGAGATACCTA
CAGCGTGAGCTATGAGAAAGCGCCACGCTTCCCGAAGGGAGAAAGGCGGACAGGTATCCGGTAAGCGGCAG
GGTCGGAACAGGAGAGCGCACGAGGGAGCTTCCAGGGGGAAACGCCTGGTATCTTTATAGTCCTGTCGGGTT
TCGCCACCTCTGACTTGAGCGTCGATTTTTGTGATGCTCGTCAGGGGGGCGGAGCCTATGGAAAAACGCCAGC
AACGCGGCCTTTTTACGGTTCCTGGCCTTTTGCTGGCCTTTTGCTCACATGTTCTTTCCTGCGTTATCCCCTGATT
CTGTGGATAACCGTATTACCGCCTTTGAGTGAGCTGATACCGCTCGCCGCAGCCGAACGACCGAGCGCAGCGA
GTCAGTGAGCGAGGAAGCGGAAGAGCGCCTGATGCGGTATTTTCTCCTTACGCATCTGTGCGGTATTTCACAC

CGCATATATGGTGCACTCTCAGTACAATCTGCTCTGATGCCGCATAGTTAAGCCAGTATACACTCCGCTATCGCT
ACGTGACTGGGTCATGGCTGCGCCCCGACACCCGCCAACACCCGCTGACGCGCCCTGACGGGCTTGTCTGCTC
CCGGCATCCGCTTACAGACAAGCTGTGACCGTCTCCGGGAGCTGCATGTGTCAGAGGTTTTCACCGTCATCACC
GAAACGCGCGAGGCAGCTGCGGTAAAGCTCATCAGCGTGGTCGTGAAGCGATTCACAGATGTCTGCCTGTTC
ATCCGCGTCCAGCTCGTTGAGTTTCTCCAGAAGCGTTAATGTCTGGCTTCTGATAAAGCGGGCCATGTTAAGGG
CGGTTTTTTCCTGTTTGGTCACTGATGCCTCCGTGTAAGGGGGATTTCTGTTCATGGGGGTAATGATACCGATG
AAACGAGAGAGGATGCTCACGATACGGGTTACTGATGATGAACATGCCCGGTTACTGGAACGTTGTGAGGGT
AAACAACTGGCGGTATGGATGCGGCGGGACCAGAGAAAAATCACTCAGGGTCAATGCCAGCGCTTCGTTAAT
ACAGATGTAGGTGTTCCACAGGGTAGCCAGCAGCATCCTGCGATGCAGATCCGGAACATAATGGTGCAGGGC
GCTGACTTCCGCGTTTCCAGACTTTACGAAACACGGAAACCGAAGACCATTCATGTTGTTGCTCAGGTCGCAGA
CGTTTTGCAGCAGCAGTCGCTTCACGTTCGCTCGCGTATCGGTGATTCATTCTGCTAACCAGTAAGGCAACCCC
GCCAGCCTAGCCGGGTCCTCAACGACAGGAGCACGATCATGCGCACCCGTGGCCAGGACCCAACGCTGCCCG
AGATGCGCCGCGTGCGGCTGCTGGAGATGGCGGACGCGATGGATATGTTCTGCCAAGGGTTGGTTTGCGCAT
TCACAGTTCTCCGCAAGAATTGATTGGCTCCAATTCTTGGAGTGGTGAATCCGTTAGCGAGGTGCCGCCGGCTT
CCATTCAGGTCGAGGTGGCCCGGCTCCATGCACCGCGACGCAACGCGGGGAGGCAGACAAGGTATAGGGCG
GCGCCTACAATCCATGCCAACCCGTTCCATGTGCTCGCCGAGGCGGCATAAATCGCCGTGACGATCAGCGGTC
CAGTGATCGAAGTTAGGCTGGTAAGAGCCGCGAGCGATCCTTGAAGCTGTCCCTGATGGTCGTCATCTACCTG
CCTGGACAGCATGGCCTGCAACGCGGGCATCCCGATGCCGCCGGAAGCGAGAAGAATCATAATGGGGAAGG
CCATCCAGCCTCGCGTCGCGAACGCCAGCAAGACGTAGCCCAGCGCGTCGGCCGCCATGCCGGCGATAATGG
CCTGCTTCTCGCCGAAACGTTTGGTGGCGGGACCAGTGACGAAGGCTTGAGCGAGGGCGTGCAAGATTCCGA
ATACCGCAAGCGACAGGCCGATCATCGTCGCGCTCCAGCGAAAGCGGTCCTCGCCGAAAATGACCCAGAGCG
CTGCCGGCACCTGTCCTACGAGTTGCATGATAAAGAAGACAGTCATAAGTGCGGCGACGATAGTCATGCCCCG
CGCCCACCGGAAGGAGCTGACTGGGTTGAAGGCTCTCAAGGGCATCGGTCGAGATCCCGGTGCCTAATGAGT
GAGCTAACTTACATTAATTGCGTTGCGCTCACTGCCCGCTTTCCAGTCGGGAAACCTGTCGTGCCAGCTGCATT
AATGAATCGGCCAACGCGCGGGGAGAGGCGGTTTGCGTATTGGGCGCCAGGGTGGTTTTTCTTTTCACCAGTG
AGACGGGCAACAGCTGATTGCCCTTCACCGCCTGGCCCTGAGAGAGTTGCAGCAAGCGGTCCACGCTGGTTTG
CCCCAGCAGGCGAAAATCCTGTTTGATGGTGGTTAACGGCGGGATATAACATGAGCTGTCTTCGGTATCGTCG
TATCCCACTACCGAGATATCCGCACCAACGCGCAGCCCGGACTCGGTAATGGCGCGCATTGCGCCCAGCGCCA
TCTGATCGTTGGCAACCAGCATCGCAGTGGGAACGATGCCCTCATTCAGCATTTGCATGGTTTGTTGAAAACCG
GACATGGCACTCCAGTCGCCTTCCCGTTCCGCTATCGGCTGAATTTGATTGCGAGTGAGATATTTATGCCAGCC
AGCCAGACGCAGACGCGCCGAGACAGAACTTAATGGGCCCGCTAACAGCGCGATTTGCTGGTGACCCAATGC
GACCAGATGCTCCACGCCCAGTCGCGTACCGTCTTCATGGGAGAAAATAATACTGTTGATGGGTGTCTGGTCA
GAGACATCAAGAAATAACGCCGGAACATTAGTGCAGGCAGCTTCCACAGCAATGGCATCCTGGTCATCCAGCG
GATAGTTAATGATCAGCCCACTGACGCGTTGCGCGAGAAGATTGTGCACCGCCGCTTTACAGGCTTCGACGCC
GCTTCGTTCTACCATCGACACCACCACGCTGGCACCCAGTTGATCGGCGCGAGATTTAATCGCCGCGACAATTT
GCGACGGCGCGTGCAGGGCCAGACTGGAGGTGGCAACGCCAATCAGCAACGACTGTTTGCCCGCCAGTTGTT
GTGCCACGCGGTTGGGAATGTAATTCAGCTCCGCCATCGCCGCTTCCACTTTTTCCCGCGTTTTCGCAGAAACG
TGGCTGGCCTGGTTCACCACGCGGGAAACGGTCTGATAAGAGACACCGGCATACTCTGCGACATCGTATAACG
TTACTGGTTTCACATTCACCACCCTGAATTGACTCTCTTCCGGGCGCTATCATGCCATACCGCGAAAGGTTTTGC
GCCATTCGATGGTGTCCGGGATCTCGACGCTCTCCCTTATGCGACTCCTGCATTAGGAAGCAGCCCAGTAGTAG
GTTGAGGCCGTTGAGCACCGCCGCCGCAAGGAATGGTGCATGCAAGGAGATGGCGCCCAACAGTCCCCCGGC
CACGGGGCCTGCCACCATACCCACGCCGAAACAAGCGCTCATGAGCCCGAAGTGGCGAGCCCGATCTTCCCCA
TCGGTGATGTCGGCGATATAGGCGCCAGCAACCGCACCTGTGGCGCCGGTGATGCCGGCCACGATGCGTCCG
GCGTAGAGGATCGAGATCTCGATCCCGCGAAATTAATACGACTCACTATAGGGGAATTGTGAGCGGATAACA
ATTCCCCTCTAGAAATAATTTTGTTTAACTTTAAGAAGGAGATATACCATGGGCCATCATCATCATCATCATCAT
CATCATCACAGCAGCGGCCATATCGAAGGTCGTCATATGAGTGCCCATCTGATTAAGATTGTTACCCAGGAAG
GCGATAGTGTTTGCTTTGATTGCTTTGAAGATGAAGATATTGTGAGTGCCGGTCTGCGTCAGGAAATCTATCTG
ATGACCAGCTGTCGCGAAGGTGGCTGCGCCACCTGTAAAGGTCTGTGCACCGATGGCGATTATGAACTGGGT
AAAGTGAGTAGTCAGGCCCTGCCGACCGAAGAAGAAGAAAATGGCTATGTTCTGCTGTGCCGTTGTTATCCGA

CCAGTGATATGGTGGTTGAAGTTCCGTATACCTATGATCGTATTAGCTTTAGTCCGGTTGGCATTAGCGTTGAA
GCAGAAATTGTGGAACTGGCCAAAGTTAGCAGTAATGTTATGAAACTGCAGCTGCAGAGTCCGCCGGATGAA
CTGAAAATTCGTTTTGATGCCGGCCAGTTTTTCGATCTGGAAATTCCGGGCACCACCACCACCCGTAGTTATAG
CCCGGCAAATATTAGTAATGATCGCGGTGAACTGGAATTTCTGATTCGTATTGTGGATAATGGCAAATTTTCTG
GTTGGCTGCAGAATCAGGCACATGTGGGTCAGAAAATTAATGTTAAAGGTCCGAGTGGCATTTTTGGTCTGAA
AGAAAATGGCTTTACCCCGCGCTATTTTGTGGCCGGCGGCACCGGCCTGGCACCTATTCTGAGCATGGTTCGCC
GTATGCGCGAATGGGAAGAACCGCAGACCAGTATTATCTATTTTGGCGTTAATACCGAAGCAGAAGTTTTCTA
TGCAGAAGAACTGAAACGTCTGGAAAGTGAAATGCCGAATCTGGGCATTCGCATTTGCGTTTGGAAAGCCAGT
GATGATTGGAGTGGTGAAAAAGGTAGCGTGGTTGATGTGCTGCGCCGTGATCTGCAGGGCGGTGGCGTTACC
CCGGATCTGTATCTGTGCGGTCCGCCGGGTATGGTGGATGCAGTGTATGCCGTTTGTGCCGAAGCAGGTATTG
CACAGAATAAGATTTTTCTGGAAAAGTTTCTGCCGAGCGTGAGCTAAGGATCCGGCTGCTAACAAAGCCCGAA
AGGAAGCTGAGTTGGCTGCTGCCACCGCTGAGCAATAACTAGCATAACCCCTTGGGGCCTCTAAACGGGTCTT
GAGGGGTTTTTTGCTGAAAGGAGGAACTATATCCGGATATCCCGCAAGAGGCCCGGCAGTACCGGCATAACC
AAGCCTATGCCTACAGCATCCAGGGTGACGGTGCCGAGGATGACGATGAGCGCATTGTTAGATTTCATACACG
GTGCCTGACTGCGTTAGCAATTTAACTGTGATAAACTACCGCATTAAAGCTTATCGATGATAAGCTGTCAAACA
TGAGAA
